# Spirit of the English Periodicals, and Notices of English Medical Literature

**Published:** 1836-10-01

**Authors:** 


### Periscope;

OR,

### CIRCUMSPECTIVE REVIEW.

"Ore trahit quodcunque potest, atque addit acervo."

### I.

### Spirit of the English Periodicals, and Notices of English Medical Literature.

### On the Use of Tartar-Emetic, combined with Opium, in certain Varieties of Delirium, occurring at an advanced Stage of Continued Fever. By R. G. Graves, M.D.

In the Numbers for May and July of our Dublin contemporary, there are two extensive papers on the above subject. The first formed the substance of two lectures in the London Med. and Surg. Journal for May, 1835; but the subject is now much enlarged, and revised.

Our able author remarks, in limine, that delirium tremens in the young and sober requires very different treatment from that which is necessary in the aged, debilitated, and intemperate. Between these two extremes there are many grades and shades, requiring modifications of practice that must necessarily be guided by the skill and attention of the physician.

"Thus, some must be treated rather actively, on the antiphlogistic plan at first, and immediately afterwards opiates may be used with advantage; while in others, opiates cannot be given alone at any period of the disease, so prominently marked are the symptoms of cerebral congestion; and yet these cases cannot be cured without narcotics. How, then, are they to be exhibited? Do we possess any medicine capable of modifying and diminishing their injurious effects when given where cerebral congestion exists? Undoubtedly we do; tartar-emetic will often accomplish this desirable object, and in delirium tremens, the value of its combination with opium is recognized by every practitioner of experience. Tartar-emetic, boldly exhibited, often is itself our sheet-anchor in delirium tremens, especially when the evidence of active determination to the head is undoubted. Then tartar-emetic alone, in repeated doses, often powerfully contributes to produce tranquillity and sleep; but there are other, more mixed cases, where we cannot cure without adding opium, sometimes in larger, sometimes in smaller quantities, to the solution of tartar-emetic; and so it is with the delirium and sleeplessness so often met with in continued fever. Every one is acquainted with the indications denoting the propriety of adopting the antiphlogistic practice when these symptoms make their appearance in the commencement of fever. Then the lancet, leeches, purgatives, cold applications to the head, and, finally, repeated doses of tartar-emetic, tend powerfully to reduce vascular action, and diminish the violence of symptoms depending on cerebral congestion and excitement. Here the lancet and tartar-emetics are our best opiates, our best restoratives of tranquillity and sleep. As the fever progresses, and when we have arrived at a more advanced stage of the disease, when petechiæ or maculæ make their appearance on the skin, and symptoms of general debility, announcing the typhoid type, begin to predominate, then we must proceed with more caution, even though our patient is totally deprived of sleep, and is violently delirious. The lancet cannot now be resorted to; leeches, indeed, may be applied, but their effects must be carefully watched, as the patient will not bear copious depletion of any sort; tartar-emetic may, nevertheless, still be given boldly, and still will be found to answer our expectations. But if we have to contend with want of sleep and delirium at a still more advanced period of fever, we now often recognize that very combination of symptoms, the union of general debility and cerebral congestion, which, in certain varieties of delirium tremens, we have seen so successfully treated with tartar-emetic and opium; who will refuse to acknowledge the similarity between these cases of fever delirium and many varieties of delirium tremens? are there not in both, the same tremor and subsultus of the extremities; the same trembling of the tongue when the patient endeavours to put it out; the same starting and sleeplessness; the same rambling, delirium or incoherence, combined nevertheless with the power of answering rationally when spoken to; the same character of the mental wandering, for in both they are extremely apt to rave as if employed in their ordinary occupations, and as if surrounded with their usual associates; in short, can any greater resemblance exist between two diseases arising from the operation of remote causes so different? We need not, therefore, be surprised, at finding the same treatment applicable to both."

The discovery of this practice in the advanced stages of spotted fever, Dr. Graves claims as his own, and we do not know any thing to the prejudice of this claim. If this practice has "not only diminished the rate of mortality in a remarkable manner in our hospitals, but has been the means of saving many of his friends and pupils," Dr. G. may certainly congratulate himself on the discovery. The following is one of the formulæ:

℞. Antimon. tart. .... gr. iv.

Tinct. opii........ fl. ʒj.

Misturæ camphoræ, ℥viij.

Misce, et capiat coch. j. mag. secundâ quâque horâ.

This medicine vomited the young gentleman to whom it was given; "but the success was very striking." He threw up a quantity of bile, and after the third or fourth dose he fell asleep, and awoke refreshed. As the case itself is illustrative and interesting, we shall here quote it.

"Shortly after the commencement of our present session, Mr. Cookson, a pupil at this hospital, and remarkable for his diligent attention to clinical pursuits, caught fever while attending our wards, in which many cases of the present epidemic were then under treatment. His fever was of an insidious nature, not characterised by any prominent symptom, not exhibiting any local disease to combat, or any tendency to crisis. For the first seven or eight days, with the exception of headach, which was much relieved by leeching, he seemed to be going on very well; his skin was not remarkably hot; he had no great thirst, nausea, or abdominal tenderness; his pulse was only 85; and he had sweating, which was followed by some relief. About the eighth or ninth day the pulse rose, and he began to exhibit symptoms of an hysteric character. Now, in every case of fever, where symptoms resembling those of hysteria come on, you should be apprehensive of danger. I do not recollect having ever met with a single case of this kind which did not terminate in nervous symptoms of the most formidable nature. I prescribed at the time the usual anti-hysteric medicines, but without any hope of doing good, knowing that these symptoms were only precursory to something worse. I also, as a precautionary measure, had leeches applied to his head. The fever went on, the headach became more intense, he grew nervous and sleepless, and fell into a state of great debility. On the fourteenth day of fever his tongue was black and parched, his belly tympanitic; he was passing every thing under him unconsciously; he had been raving for the last four days, constantly attempting to get out of bed, and had not slept a single hour for five days and nights. Dr. Stokes, with his usual kindness had given me the benefit of his advice and assistance during Mr. Cookson's illness, and we had tried every remedy which experience could suggest. Blisters had been applied to the nape of the neck, the head kept cool by refrigerant lotions, the state of the belly attended to, and, as we perceived that the absence of sleep was a most prominent and distressing symptom, we had been induced to venture on the cautious use of opium. It was first given in the form of hydrarg. c̄ cretâ with Dover's powder, with a view of relieving the abdominal symptoms, as well as procuring sleep. This failing in producing the desired effect, we gave opium in the form of enema, knowing its great power in the delirium which follows wounds and other injuries. This was equally unsuccessful with the former. He still was perfectly sleepless. We came again in the evening, and as a last resource, prescribed a full dose of black-drop, and left him with the conviction, that if this failed he had no chance of life. On visiting him next morning at an early hour, we were highly mortified to find that our prescription had been completely unsuccessful; he had been more restless and delirious than ever. Here was the state in which we found him on entering his chamber at eight o'clock in the morning, on the 15th day of his fever. He had universal tremors and subsultus tendinum, his eye was suffused and restless, he had been lying for some days entirely on his back, his tongue was dry and black, his belly tympanitic, his pulse 140, quick and thready, his delirium was chiefly exhibited in short broken sentences and in a subdued tone of voice, and it was now six days and nights since he had slept. Here arose a question of great practical importance. How was the nervous agitation to be calmed and sleep produced? Blisters to the nape of the neck, cold applications, and purgatives had failed; opium in various forms had been tried without the slightest benefit; if sleep were not speedily obtained he was lost. At this emergency, a mode of giving opium occurred to me which I had never thought of before in such cases. Recollect what his symptoms were at this period: quick failing pulse, black, dry, tremulous tongue, some tympanitis, excessive prostration of strength, subsultus tendinum, extreme nervous agitation, constant muttering, low delirium, and total sleeplessness. I said to Dr. Stokes that I wished to try what effects might result from a combination of tartar-emetic and opium; I mentioned that I had given it in cases of delirium tremens with remarkable success, and thought it worthy of trial under the circumstances then present. Dr. Stokes stated in reply that he knew nothing with respect to such a combination, as adapted to the case in question, that he had no experience to guide him, but that he would yield to my suggestion. We therefore prescribed a combination of tartar-emetic and laudanum in the following form, which is that in which I generally employ these remedies in the treatment of delirium tremens."

Another case of another pupil is detailed by Dr. Graves. On the 17th day of the fever, he had brown tongue and dry—abdominal tenderness—red and ferretty eyes—no sleep—constant muttering delirium, assuming the form of delirium tremens—with subsultus tendinum, &c. Dr. Graves directed the combination of tartar-emetic and laudanum to be given. He had only taken two doses when calmness took place, with relief of all the symptoms. He fell into a profound sleep, and awoke refreshed. The mixture was continued every four hours, with increasing benefit. In about a week he was able to sit up in bed, and seven days afterwards he left the hospital.

Another case is reported—that, also, of a pupil—in which the practice in question was equally successful. Since the lecture in which these cases were first detailed, many cases have been treated on the same plan by Dr. Graves, and are related in the Dublin Journal. To this source we must refer our readers for further particulars. We shall conclude this notice with the following remarks of our very talented contemporary.

"One fact connected with the cases just related is very striking, viz. the small quantity of laudanum which, in most of them, was sufficient to induce sleep; a circumstance only to be accounted for by the presence of the tartar-emetic, which no doubt exerts, when given in duly regulated doses, a powerfully tranquillizing effect on the nervous system. It is also deserving of remark, that the medicine very seldom gives rise to any of the unpleasant symptoms that so frequently arise when opium alone, or any of its preparations, are given with a view of producing sleep at an advanced period of fever. The addition of one ounce of mucilage, and one ounce of simple syrup to the mixture, seems to render it less likely to disagree with the stomach. Towards the termination of fever, it not unfrequently happens that a sudden or gradual determination of blood to the head arises, and which requires a repetition of a modified system of antiphlogistic treatment, aided by blisters. This state, I have reason to believe, may be often prevented from occurring, by a timely attention to procuring sleep; for a patient in fever, who has passed several sleepless nights, is on the verge of cerebral congestion or inflammation, as is testified by headach, wandering, and the redness of the conjunctiva. Here it is that the treatment I recommend is so advantageous, when timely applied; for if it be deferred until cerebral inflammation has set in, opium in any shape is worse than useless.

I have notes of several other cases, equally strong, in favour of the utility of tartar-emetic and opium in the advanced stage of fever, but think it unnecessary to bring them forward, as the above seem sufficient for my present purpose. The particular state of the nervous system to which this combination of remedies is best adapted, may occur, along with other symptoms produced by functional or organic lesions of various organs, and which prevent it from producing the wished-for beneficial result. Thus when the belly is tense and swollen, this remedy will generally fail; but I think that I am warranted in asserting that in fevers, properly treated from the first, tympanitis may commence, but will never become considerable; for, if the attention of the practitioner be applied to this symptom the moment it begins to show itself, he can in most cases succeed in arresting its progress. I have likewise seen several cases of fever, where I expected benefit from the tartar-emetic and opium, and in which no good result followed the exhibition of these medicines: such failures must always occur with respect to every remedy we apply in disease, but they do not invalidate the evidence of facts, such as I have brought forward in proof of their frequent utility. In connexion with this subject, I beg leave to draw the attention of practitioners to the occurrence of *delirium traumaticum* in fevers, in consequence of the irritation produced by blisters, a species of delirium apt to be mistaken, especially in children, for the delirium ushering in hydrocephalus. It is unnecessary to do more than advert to this subject, as I have spoken of it at some length in the lectures before referred to. To conclude, it is right to remark, that the relative proportions of tartar-emetic and laudanum in the mixture must be varied according to circumstances.

When congestion of the brain is known to exist, or is feared, the tartar-emetic must not fall short of four grains in the eight ounces, while the laudanum should not exceed half a drachm; but where nervous symptoms predominate, the laudanum may amount to one drachm, and the tartar-emetic to two grains: no general rule, however, can be laid down, *and the practitioner must in all cases watch the effects of this medicine, from hour to hour,* until he ascertains whether it agrees with the patient or not. Where a life is at stake, we must spare no pains, and must not reject a remedy because its powers render it an instrument of good or evil, according as it is administered carefully or otherwise."

We fully concur in the justice of these last remarks; but we put it to the judgment of Dr. Graves, who is himself deeply immersed in private practice, how it is possible to watch these cases in the manner described, except in hospitals, where house pupils can be constantly in attendance? Medical practitioners must live—hardly enough, God knows—and if all their attention is to be devoted to a single patient, from hour to hour, their other patients will murmur, and they themselves will starve! These important "instruments of good or evil," therefore, must be administered in public institutions chiefly—and only on a limited scale in private life.

---

### Gun-shot Wound of the Face and Neck, with Rupture of the Common Carotid.

The following case has been communicated to us by our able friend Mr. Guthrie. It was mentioned by him to his class in a clinical lecture which he delivered at the Westminster Hospital. Appended to it are a few remarks by the lecturer. The case does much credit to Mr. Alcock, who had the charge of it at Oporto, and who transmitted the particulars to Mr. Guthrie. Mr. Alcock is now employing his talents with the British Legion in Spain, and is likely to prove a worthy addition to the already honorable list of distinguished army surgeons.

"S. Brannigan, private of the Scotch Fusiliers in the service of Portugal, was admitted into Hospital March 4th, 1833, a musket-ball having passed through his neck and face during the action which took place on that day at Oporto.

My attention was called to him on the field a few minutes after he received the wound. He was then lying on the ground insensible, and had evidently bled profusely. I found the ball had entered the right cheek on a level with the alveolar processes of the two last molar teeth, severely fracturing the lower jaw, and passing downwards, made its exit from the neck on the opposite side above the clavicle, grazing the shoulder afterwards. I dressed him lightly, all bleeding having stopped, and had him at once conveyed to the hospital.

On the 6th I dressed him myself—he was perfectly sensible, though there still remained some evidence of the severe cerebral shock. The neck and face were largely swelled. An enema of the sulphate of magnesia had been administered soon after his admission—cold applications over the light dressing, and a few spoonfuls of barley-water and arrow-root formed the whole of the treatment adopted at this time. He experienced the greatest difficulty in swallowing.

On the 7th, the discharge from the neck became profuse, and on the 8th, the sloughs were separating, and a great portion of that on the neck came away. On the 9th, I found there had been a little bleeding from *both* the wounds. On the 11th, hæmorrhage to a considerable extent took place from the wound in the neck, the blood seemed partly venous and partly arterial, and was apt to recur whenever he coughed, to which he was constantly liable from the pressing sense of uneasiness in the throat.

On the 12th, I found two or three ounces of blood, principally arterial, had been lost during a coughing fit—the discharge from the neck continued exceedingly copious. During the 13th and 14th, at intervals of a few hours, whenever by his restlessness or coughing he shook himself, bleeding recurred, sometimes from both wounds, sometimes from the neck, and sometimes from the cheek. Although the quantity lost each time seldom exceeded three ounces, still its constant recurrence at this period had seriously affected him. His pulse became quick and rather wiry, and he suffered from great restlessness. He was moreover exceedingly obstinate, and refused to attempt to pass any thing in the shape of medicine down his throat, and indeed the violence with which the cough sometimes succeeded any attempt to swallow, rendered it of doubtful expediency. His bowels were left open by enemas.

From the 14th to the 15th scarcely any hæmorrhage took place, but in the afternoon of that day, there was rather a profuse hæmorrhage from the cheek, and apparently from the labial artery; pressure on the ramus of the jaw, with cold applications eventually stopped it. On the 16th there was no further bleeding—the pulse had become very languid. He had passed however a quiet night—an anodyne draught formed of tr. opii, tr. digitalis, and mist. camphoræ, was ordered to be given thrice during the 24 hours, if he could swallow it without violence.

At this period the necessity of taking more active measures to prevent the constant recurrence of bleeding, which was evidently wearing him out, was pressed more strongly upon me in proportion as all hopes of the stoppage of the bleeding-vessels by the healing process of nature weakened and failed. Nevertheless there were many contradictory and embarrassing circumstances in the case, which rendered it a matter of doubt how far any operation might be successful. It was difficult to believe that the carotid itself was wounded, for it was contrary to all our experience of wounded arteries of that calibre, that during twelve days no fatal nor indeed very large hæmorrhage should take place. Add to this the bleeding sometimes from one, and sometimes from both wounds, and I think it will be allowed that this formed a case of no ordinary difficulty. The surgeons of H.M.S. the Nautilus and Orestes, then in the Douro, as also Dr. Jebb, an experienced British staff surgeon, had very kindly taken all the circumstances of the case into consideration, and given me their opinions. They felt the same difficulties I have described in coming to any accurate conclusion on the precise source or sources of the hæmorrhage, and did not conceive, under that uncertainty, any operation justifiable. It was on this day, the 16th, a little after noon, as I was preparing to leave the hospital, that an orderly came to me in great haste to say that the hæmorrhage had returned with greater violence than usual. I hastened to him, nevertheless he had already lost several ounces of blood, and finding that pressure below on the common carotid artery effectually stopped the bleeding, I determined immediately to cut down upon the trunk of the carotid, and place a ligature on it, believing the hæmorrhage, if it came not direct from it, must at least arise from some large branch, the superior thyroid or lingualis, just as they were given off. And, at all events, it seemed to me the only chance left of preventing his death by hæmorrhage. There was not time to secure the able advice and assistance of the experienced friends I have mentioned, and therefore with those of my own medical officers who happened to be on the spot, I had him placed on a bed with his head to the light, his shoulders and head gently raised. An incision over the inner edge of the mastoid muscle, from about the level of the cricoid cartilage to within a short distance of the sternum, discovered the edge of the muscle; but I found it so firmly bound down on every side by lymph and hardened cellular tissue, that neither with my nail or the handle of my scalpel could I move it a single line, or separate it from the parts below. This separation was effected therefore with the edge of the scalpel, but instead of finding the smooth cervical fascia, and marking the omo-hyoideus passing across, there was nothing but diseased cellular tissue, hardened and altered alike in colour and texture, by the lymph thrown out in the inflammatory process that had been set up for so many days. I felt the artery feebly pulsating some time before I could see it, in which I succeeded only after removing a large coagulum of fibrine, by which the artery was laid bare at a considerable depth from the surface, in the course of the ball which I had cut across, and with a lacerated opening into it, more than a quarter of an inch in length. The vein also appeared to me to be lacerated. I endeavoured to pass a blunt needle under the artery, but found it so firmly attached to the diseased parts in which it was embedded, that nothing but the point of the knife could detach it. On the side next the trachea it was equally firmly bound, and in the endeavour to separate it, a longitudinal incision of about a quarter of an inch was made in the trachea. A little blood escaped at the same moment, the air was violently expired for an instant, and to all appearance he sank, and was in articulo-mortis. He seemed to be dying without an effort or a struggle. For an instant I was myself paralysed from the conviction of the hopelessness of any attempt to save or restore him. A second moment's reflection, however, convinced me of the expediency of concluding the operation. I therefore immediately passed a needle through the wounded part of the trachea, and brought the edges together by a suture. The artery was next secured above and below the ruptured portion. The integuments were brought together by strapping and suture; and in a very few minutes I had the pleasure and satisfaction of seeing him rally so as to be able to speak and answer the question put to him. There was not the slightest oozing of blood, and he breathed perfectly freely. The cold lotion was continued.

With respect to the subsequent treatment, it is only necessary here to remark, that no untoward symptom was observed in the neck up to the period of its being entirely healed. Some sensorial disturbance with fever came on a few days after the operation—on his recovery from that the neck rapidly healed.

His wound in the cheek with fractured jaw was much less manageable—he was of a scrophulous habit, and sores and abscesses formed successively on that side about the glands of the cheek, jaw, and neck; and on my proceeding to Santarem with the British, he was forwarded to Lisbon, and only came under my charge again a very short time before I left Portugal, at which time, as he was about to be sent to England, I gave him a letter to Mr. Guthrie, to whose instructions on the subject of wounded arteries and hæmorrhage I owe that feeling of confidence in my own power over them, which, not only in this case, but in a larger number of amputations and wounded arteries, enabled me to undertake their performance with scarcely any assistance—under circumstances too, where, under more timid council, I might have felt hesitation in attempting to operate even with the best and most efficient assistants. There are many conclusions highly interesting which may be drawn from the facts of this case, but these I defer to a future occasion."

Since he has been in this hospital, I have cut down on the upright or ascending process of the jaw, and removed as you saw a considerable portion in two pieces of the ramus and condyloid process with the articulating surface. This has given him great relief, but the openings have not yet healed, and there is a small portion of bone to come away. In this case the jaw has not suffered from necrosis apparently, but the dead bone has been merely deprived of life and slowly separated from the living. He cannot open his mouth, from the consolidation of parts on the side of the jaw and neck, but he can protrude his tongue, and when he does so, it turns out at the right corner of the mouth; the muscles on the left side being paralysed, and evidently not in action, arising, I presume, from some injury committed on the ninth nerve, I should suppose either by the ball or in the operation. The senses of taste and feeling on this side seem to be unimpaired, certainly as to feeling, and I believe so as to taste, as I cannot make out any defect on trial.

---

### Pathology puzzled.

A medical officer of the army lately came under the care of Drs. Theodore Gordon, Mr. Guthrie, and Dr. J. Johnson. He was about 53 years of age, and had served in the Peninsular war, where he was affected with ague, and, as he believed, liver-complaint. He had afterwards served at the Cape, where he was subject to bilious affection and dyspepsia. His regiment was stationed at Hounslow, where he became affected with distressing nausea and vomiting after taking food, with rapid emaciation, and some yellowness of the skin. He was brought to town, and carefully attended to by the above-mentioned gentlemen. There was tenderness at the epigastrium, but no distinct hardness in the region of the pylorus. There was a loud bruit de soufflet, and considerable impulsion in the cardiac region. The pulse was seldom under 100 in the minute—the emaciation extreme—nausea or vomiting every day after food, with ejection of bile—irritable state of bowels—furred tongue—sallow and yellow tinge of complexion—despondency and irritability. The diagnosis was obscure. The heart, the liver, and the stomach, especially the pylorus, were suspected of being organically diseased. The chief attention was, of course, directed to the stomach. Leeches, blisters, effervescing draughts were prescribed; but the unfortunate patient gradually sank and died.

He was carefully examined by Mr. Hancock, a Demonstrator of Anatomy, in the presence of several medical gentlemen, among whom were Dr. Gordon, Mr. Guthrie, Mr. Henry J. Johnson, &c. The report was, that no organic disease existed in any part of the body, and that the mucous membrane of the stomach *only* was found to be phlogosed. Dr. Johnson was not able to attend the dissection, but there can be no doubt of the accuracy of the investigation. He confesses that he never was more surprised than by this result of a *post-mortem* inspection. He fully expected that a large amount of organic disease would have been discovered. Such, however, was not the case. The event is worth recording, as a lesson not to be too confident in our pathological diagnoses! Would that every subject could be accurately examined and faithfully narrated!

---

ENURESIS.

A case of inability to retain the urine is related by Dr. Roots in the 4th number of St. Thomas's Hospital Reports. The patient was a young female, aged 16 years, who had recently menstruated for the first time. The enuresis had continued, more or less, for five years. She could only retain her water when sitting, and perfectly quiet. She had complete command over the rectum. She was ordered a dose of castor oil, and then to take 15 drops of the tinct. lyttæ every six hours. A blister to the sacrum. She had no involuntary discharge afterwards. The tinct. lyttæ, however, was increased to twenty minims every six hours for a few days. She was then discharged cured.

We believe the lytta to be the best remedy in such cases, and it may be taken to a very considerable extent. We have exhibited up to three or four drachms per diem, without any bad effects. A slight strangury is the signal for lessening the dose or discontinuing the medicine.

---

CELSUS. By ALEXANDER LEE, A.M., Surgeon. With explanatory Notes, &c. &c. for the Use of Students. In Two Volumes. Vol. II. Cox, St. Thomas Street, Borough. 1836.

Of all the ancient medical authors, Celsus is, incomparably, the most interesting, both as to elegance of language and importance of matter. If Celsus or Hippocrates were to be lost to the world, it were infinitely better to let the Father of Physic go to the flames, than his Italian successor. In all the old authors there is a prodigious deal of useless or unintelligible rubbish mixed up with what is good or true. In Celsus, there is hardly a paragraph, from beginning to end, that we could wish to be cancelled. Of all the compilers that ever lived, Celsus was probably the most judicious in selecting the grain from the chaff. He has condensed all that was worth knowing up to his own time, and in his work will be found a good deal of all that we know up to this day. Mr. Lee has conferred no trifling advantage on students and practitioners in general, by the publication of these two volumes, with clear and accurate text, perspicuous ordo, literal, but easy translation, useful notes, and a copious index. To the student, who is now examined in Celsus, this edition will be a treasure, and to the laborious practitioner, who may not have studied this elegant medical classic, the present work will offer an easy means of going through the original without difficulty, and without reference to the Dictionary. We gave a sample of the first volume. We will now do the same from the present and concluding one.

QUOMODO AQUA HYDROPICIS EMITTATUR.

Aquam iis, qui hydropici sunt emitti oportere, alias dixi. Nunc, quemadmodum id fiat, dicendum est. Quidam autem sub umbilico, fere quatuor interpositis digitis a sinistra parte: quidam, ipso umbilico perforato, id facere consuerunt: quidam, cute primum adusta, deinde interiore abdomine inciso; quia, quod per ignem divisum est, minus celeriter coit.

Ferramentum autem demittitur magna cura habita, ne qua vena incidatur. Id tale esse debet, ut fere tertiam digiti partem latitudo mucronis impleat; demittendumque ita est, ut membranam quoque transeat, qua caro ab interiore parte finitur: eo tum plumbea aut ænea fistula conjicienda est, vel recurvatis in exteriorem partem labris, vel in media circumsurgente quadam mora; ne tota intus delabi possit. Hujus ea pars, quæ intra, paulo longior esse debet, quam quæ extra; ut ultra interiorem membranam procedat. Per hanc effundendus humor est; atque ubi major pars ejus avocata est, claudenda demisso linteolo fistula est; et in vulnere, si id ustum non est, relinquenda. Deinde per insequentes dies circa singulas heminas emittendum, donec nullum aquæ vestigium appareat. Quidam tamen etiam non usta cute, protinus fistulam recipiunt, et super vulnus spongiam expressam deligant: deinde postero die rursus fistulam demittunt (quod recens vulnus paulum diductum patitur) ut, si quid humoris superest, emittatur: idque bis ita fecisse contenti sunt.

ORDO.

QUOMODO AQUA EMITTATUR HYDROPICIS.

Dixi alias, oportere aquam emitti iis, qui sunt hydropici. Est nunc dicendum quemadmodum id fiat. Autem quidam consuerunt facere id sub umbilico, fere quatuor digitis interpositis a sinistra parte; quidam umbilico ipso perforato: quidam, cute primum adusta, deinde interiore abdomine inciso; quia, quod divisum est per ignem, coit minus celeriter.

Autem ferramentum demittitur magna cura habita, ne qua vena incidatur. Id debet esse tale, ut latitudo mucronis impleat fere tertiam partem digiti; que est ita demittendum, ut quoque transeat membranum, qua caro finitur ab interiore parte: tum plumbea aut ænea fistula est conjicienda eo, vel labris recurvatis in exteriorem partem, vel quadam mora circumsurgeante in media; ne tota possit delabi intus. Ea pars hujus, quæ intra, debet esse paulo longior quam quæ extra; ut procedat ultra interiorem membranum. Per hanc humor est effundendus: atque ubi major par ejus evocata est, fistula est claudenda linteolo demisso; et relinquenda in vulnere, si id non est ustum. Deinde per insequentes dies emittendum circa singulas heminas, donec nullum vestigium aquæ appareat. Tamen quidam, etiam cute non usta, protinus recipiunt fistulam, et deligant expressam spongiam super vulnus: deinde postero die demittunt rursus fistulam, (quod recens vulnus diductum paulum patitur) ut si quid humoris superest, emittatur; que sunt contenti fecisse id ita bis."

TRANSLATION.

THE METHOD OF PERFORMING PARACENTESIS ABDOMINIS IN DROPSICAL PERSONS.

I have already stated elsewhere, that it is necessary to draw off the water from dropsical persons. I must now describe the manner of doing it. Some have been accustomed to make *the puncture* under the umbilicus, generally about four fingers' breadth to the left: some have perforated the navel itself: some have cauterized the skin first: and afterwards made an incision through the integuments of the abdomen below; for this reason, that that which was divided by fire (*actual cautery*) united more slowly.

Now the instrument is to be introduced with great care, lest a vein (*artery*)

be divided. It ought to be of such a form, that the breadth of its point should be about the third part of a finger's breadth: and it is to be passed through the membrane also, which bounds the interior part, *i. e. abdominal cavity:* then a leaden or brazen pipe is to be inserted into it, with its lips either curved outward, or surrounded with a ring about the centre, to prevent its slipping into the abdominal cavity. The portion to be introduced should be a little longer than that without, in order that it may pass beyond the inner membrane. By this the fluid is to be drawn off: and when the major part of it has been discharged, the canula is to be closed with a bit of linen: and this is to be left in the wound, if the opening had not been previously cauterized. Then on the subsequent days about a hemina should be drawn off each time, whilst any trace of water remains. Some even withdraw the canula, although the wound had not been previously cauterized, and bind a wet sponge over the wound: next day they introduce the pipe again, which the recent wound will permit, by being drawn a little apart; so that if any fluid remain, it may be discharged: they are satisfied when this is done twice."

---

### CONTRIBUTIONS TO PATHOLOGY. By W. SMITH, Esq.

[Dublin Journal, July 1836.]

1. *Lesions of the Heart.* The first case narrated by Mr. Smith was that of an old woman, aged 90, who died suddenly, having previously complained only of debility. On elevating the sternum, air was seen in the cellular tissue of the mediastinum. The pericardium was greatly distended with blood, partly fluid, partly coagulated. The heart was thickly covered with fat, and was soft, pale, and flaccid. There was a small lacerated opening near the centre of the anterior part of the left ventricle. Air was extensively diffused throughout the subcutaneous tissue of the whole body; and the liver was so softened that a stream of water poured upon it, washed away its substance. The spleen and kidneys presented similar appearances. Upon removing the liver from the body, the division of the vena cava gave exit to nearly a table spoonful of clear transparent oil. Some oil was also collected from other organs.

2. The second contribution is the case of a female, aged 70, who was admitted into the Richmond Hospital an hour or two before her death. There was much fluid in the cavity of the pleura—heart flabby, and covered with fat—the surface of the blood was thickly covered with globules of limpid oil. The blood itself was thin and unhealthy, without disposition to coagulate. The abdominal viscera were healthy.

We have abridged these two cases much, because the discovery of rare specimens of pathological anatomy in the dead-house, without a knowledge of the symptoms during life, is of little value. In Mr. Smith's comments on these two cases, however, we have something more tangible. From these we shall make an extract.

"Bearing these circumstances in recollection, we may, I conceive, look upon the deposition of a pale, gelatinous fat, as indicative and the result of enfeebled powers of assimilation and deficient energy in the circulatory system; for between the two there is a constant relation, and if the presence of free oil in that system is to be considered as denoting a superabundance of venous blood, we have then another cause, which in addition to the peculiar state of the heart, we can assign to explain the occurrence of apoplectic symptoms in such cases. Cruveilhier, in his admirable plates of pathological anatomy, delineates the morbid appearances observed in a case of fatty accumulation upon the surface of the heart, combined with rupture of the left ventricle; and I may remark that they perfectly represent the appearances observed in the case of Newman, with the exception, that in Cruveilhier's case, the walls of the ventricles were hypertrophied. It is a disease of advanced life, and generally produces sudden death, either from rupture, which is almost always seated in the left ventricle, or by inducing an apoplectic state; and it is singular that conditions of the heart, so opposite as hypertrophy and atrophy with fatty degeneration, should both produce apoplectic symptoms. M'Adams, in his talented essay upon the diseases of the heart, published in the 4th vol. of the Dublin Hospital Reports, observes that the explanation of the fact will probably "be found in the reflection, that anything occasioning an undue distention of the vessels of the brain, may be followed by apoplexy. This over-distention may arise from the impulse a tergo being preternaturally strong, or on the contrary it may be the result of some obstruction in front, as that arising from a contracted arterial opening, or some state of the ventricle, incapacitating it from emptying itself with sufficient quickness to relieve the brain;" and he imagines, "that although the *quality* of the blood may have some influence, it is probable that the principal causes determining an apoplectic attack, when the heart is either actively enlarged or in a state of atrophy, are mechanical, and referrible to circumstances in the heart, directly or indirectly producing a state of *congestion* of the vascular system of the brain."

The fatty condition of the heart and the rupture of its walls depend, most probably, on the same constitutional cause.

3. *Cartilaginous Transformation of the Heart.* This was found in a lunatic, 45 years of age, long subject to epilepsy, and who died during a convulsion. The vessels of the brain were distended with dark blood—the cavity of the left ventricle of the heart was slightly enlarged, and its parietes thinned. The muscular substance had completely lost its natural colour and consistence, especially towards the apex of the ventricle. It was converted into a dense, white, firm cartilaginous structure, the division of which, with scissars, required considerable force. This alteration of structure had extended to some of the carneæ columnæ. *The auricles were hypertrophied.* In this and in similar cases, the auricles, in fact, perform, though imperfectly, the office of the ventricles.

4. *Ossification of Pericardium.* A man, aged 39, subject to cough and dyspnœa, was found, on the 26th April, labouring under general symptoms of disease of the heart—countenance bloated and livid—great dyspnœa—tympanitic abdomen—feeble pulse—distended jugulars—skin yellow. He died on the 30th. On inspection, part of the splenic capsule was found to be cartilaginous—liver hypertrophied of its yellow tissue, the general size being diminished. The lungs were gorged with blood, and even were adherent to the costal pleura. There were some bony deposits in the left pleura costalis. The pericardium was united to the heart by close and old adhesions, and around the base of the organ bony matter was deposited in considerable quantity, forming an osseous belt, nearly surrounding the organ. The average breadth of this belt was an inch. This bony girdle penetrated into the substance of the ventricles, reaching, in some places, almost to the cavities. "The parietes of the auricles were increased in thickness—in some places to a quarter of an inch."

5. *Aneurism of the ascending Aorta.* A man, aged 30, was admitted into Sir P. Dun's Hospital, with general symptoms of disease of the heart of long standing. No accurate history could be obtained, and the man died next day. On raising the sternum the pericardium was found distended so as to conceal the lungs. It adhered firmly to the heart throughout the greater part of its extent. The origin of the aorta appeared dilated, and, at the base of the heart appeared two tumors, each the size of an egg, elevating the pericardium. These two tumors formed an aneurismal sac, slightly separated by a depression corresponding to the pulmonary artery, the passage of which made a kind of groove or sulcus in the sac. The left division of the sac had encroached considerably on the left ventricle of the heart, diminishing its capacity. It was separated from the ventricle by a thin partition. The sac itself communicated with the aorta by a round opening, the size of a shilling, and situated about an inch from the semilunar valves, leading downwards and forwards into the aneurysmal cavity. The aorta, in the vicinity of the opening, was rough and unhealthy.

"This interesting case is a sufficient refutation of the opinion of Bertin, Scarpa, and other writers upon aneurism, viz. that this disease does not and cannot form in any part of the ascending aorta, because there is no cellular structure to form the sac."

There are several other instances recorded by Guthrie and others.

---

### THE SKULL OF SPURZHEIM.

The Phrenological Journal for June, contains a report, by Dr. Shurtleff, of Boston, on the skull of Dr. Spurzheim. That amiable man and enthusiastic physiologist had expressed a wish, as Gall had done before him, that his skull should not be buried with his body, but be preserved as the memento and proof of his own doctrines. It *is* preserved, in a fire-proof safe, in the hall of the Phrenological Society of Boston, and the brain of the philosopher accompanies it.

The weight of the latter is considerable—amounting to 3 pounds, 7 ozs. and 1 drachm, after being deprived as much as possible of its fluids, and divested of the dura mater.

The cranium, adapted to contain so large a brain, must of course exceed the average dimensions. The following are its exact admeasurements, taken as far as possible from anatomical points.

| | Inches. |
|---|---|
| "Greatest circumference (measured horizontally) . . . . . | $22\frac{1}{4}$ |
| — length from occipital protuberance to the frontal sinuses . | $7\frac{1}{2}$ |
| Distance from occipital protuberance to the naso-frontal articulation measured over the head . . . . . . . | $13\frac{6}{10}$ |
| — — naso-frontal articulation to superior angle of the occipital bone . . . . . . . . . . . | $7\frac{1}{10}$ |
| — — naso-frontal articulation to the anterior extremity of the sagittal suture . . . . . . . . . | $4\frac{6}{10}$ |
| — — occipital protuberance to superior angle of the occipital bone . . . . . . . . . . . | $2\frac{8}{10}$ |
| — — occipital protuberance to anterior extremity of the sagittal suture . . . . . . . . . . | $6\frac{1}{2}$ |
| Greatest breadth of skull, measured between the temporal bones, 1 inch above the orifices of the ears . . . . . | $6\frac{1}{4}$ |
| Distance from mastoid process to mastoid process . . . . . | $5\frac{6}{10}$ |
| — — ear to ear . . . . . . . . . . . . | $4\frac{1}{2}$ |
| — — — naso-frontal articulation . . . . . | $4\frac{1}{2}$ |
| — — — frontal sinuses . . . . . . . . | $4\frac{8}{10}$ |
| — — — anterior extremity of sagittal suture . . . | $5\frac{1}{2}$ |
| — — — summit of head . . . . . . . . . | $5\frac{6}{10}$ |
| — — — superior angle of occipital bone . . . . | $4\frac{4}{10}$ |
| — — — occipital protuberance . . . . . . . | $4\frac{1}{4}$ |
| — — — ear over the summit of the skull, in a vertical direction . . . . . . . . . . . | 14 |
| — — — — around the lower part of the forehead . | $11\frac{1}{2}$ |
| — — — — — back of the skull at the occipital protuberance . . . | $9\frac{3}{4}$ |
| — — parietal protuberance to parietal protuberance . . | $5\frac{1}{2}$ |

Distance between the anterior-inferior angles of the parietal bones . $5\frac{2}{10}$

Camper's* facial angle . . . . . . . . . 61 degrees."

There appear to be some slight discrepancies between these measurements and those taken in Edinburgh from the cast. But they are comparatively trivial, and our author assures us that he was exceedingly particular.

The substance of the skull is compact, with very little diploe. On the internal surface, the digital impressions resulting from the convolutions of the brain are unusually well marked.

There are some remarks on the individual bones of the cranium, to which we do not think it necessary to allude. Our readers, whether phrenologists or not, will glance with some interest at the dimensions of a skull, from whose recesses issued the most amiable, sincere, and eloquent advocacy of the science that its owner loved.

---

### Mr. Middlemore on Pterygium.

In our Number for January last, we gave a full notice of Mr. Middlemore's essay on the above disease, which analysis has been copied into the Encyclographie des Sciences Medicales, and other foreign periodicals. The author has written to us respecting an omission which he deems of importance, as relating to the "precise seat of the pterygial production, and the mode in which it acquires an almost complete covering from the conjunctiva." The following is the omitted paragraph, which we now insert.

"In the first place, there will be seen a thin, semi-transparent layer, immediately beneath the conjunctiva—in the subconjunctival cellular membrane—in which bloodvessels are situated, which do not divide and ramify extensively, but pass onwards towards the cornea, in a direction nearly parallel with each other. This deposition increases, until, in the worst cases, it becomes a thick red mass, having a somewhat triangular figure, a flattened surface, and fibrous aspect, much resembling a small muscle. The conjunctiva becomes raised and attenuated, so as to be expanded upon its front surface, and reflected on each side upon its posterior aspect, whence it is again reflected upon the globe of the eye; so that it may be raised entirely from the sclerotica on each side; but, in the middle of its under surface, it is always adherent more or less extensively—it is, in fact, adherent, in consequence of the reflexion of the conjunctiva, on each side of its posterior surface, to the globe of the eye. Hence it may be remarked, that a probe passed behind a common fleshy pterygium, will enter a sort of *cul-de-sac*, and become obstructed at a point which will mark the line where the conjunctiva becomes reflected from the posterior aspect of the morbid production to the sclerotica."

---

### Chronic Laryngitis—Dr. Roots.

This paper appears in the 4th Number of St. Thomas's Hospital Reports. A case is first narrated, and then follow the clinical remarks.

*Case*. J. Hurley, a coal-porter, aged 26 years, was admitted on the 24th Dec. affected, for two months after exposure to cold, with severe cough, with thick yellow expectoration, succeeded by sore-throat and hoarseness. The tonsils were swelled, and the arches of the palate had a livid appearance. Pressure on the thyroid cartilage occasioned

---

* "Notwithstanding the prominence of the forehead, this measurement is taken correctly. Two causes combine to make this angle small in the head of Dr. Spurzheim; 1st, the great length of the face; and, 2d, the extra high situation of the ear. This is another fact which goes against the intellectual angle of Camper."

pain. The voice is heard in a hoarse whisper—the skin is hot and tongue coated, pulse 96. Bled to sixteen ozs. —blister to the throat—calomel purge. Antimonial wine and sulphate of magnesia every six hours.

28th. Can speak louder—has less pain on pressure—cough diminished—skin moist. 29th. Twelve leeches to the throat—one grain of calomel, and a quarter of a grain of ant. tart. every four hours. Jan. 2d. Voice decidedly better; but there is still some pain on pressing the larynx. He continued to improve, and the mercury was discontinued on the 5th. On the 15th he was considered to be well, and was discharged on the 19th of Jan. 1836.

*Clin. Rem.*—The symptoms of chronic laryngitis are commonly these:

"A hoarse, harsh cough, occasionally almost amounting to a sort of croupy, or crowing sound; the voice, too, at the same time changes: it becomes hoarse, rough, and harsh, and as the disease advances, it is often merely a hoarse harsh whisper: sometimes, indeed, it is quite sibilant, accompanied by a hissing sound; and there is also a hissing sound sometimes in respiration. The respiration is difficult and hurried. The expectoration most commonly is copious; sometimes it is only mucus, but as the disease goes on, it becomes muco-purulent, sometimes almost entirely purulent, and sometimes sanguineous, either streaked with blood, or there is occasionally expectoration, with small patches of blood. The pulse is most commonly quick; and when the disease is of long standing, it is often accompanied by great emaciation, and attended also by a considerable anxiety of countenance. Sometimes the case is attended by a very distressing perspiration. There is also commonly more or less pain in deglutition, and if you put your hand on the larynx, on directing the patient to make an effort to swallow his saliva, you notice, so far as my experience goes, that it is invariably productive of pain to a greater or less extent. I never remember to have witnessed a case of chronic laryngitis, in which this symptom was not present.

From the character of the expectoration, the emaciation, the quick pulse, and the perspiration, you will see that chronic inflammation of the larynx often simulates very closely phthisis pulmonalis. How then would you form your diagnosis? It is of the utmost importance that the diagnosis should be correct, inasmuch as the one is a case which admits of being cured, and the other, according to our present knowledge, does not. In the first place, you would judge by the degree of resonance on percussion being natural over every part of the chest, and more especially under the clavicles upon either side, the apex of the lung being the part in which, generally, tuberculous deposition commences. In addition, then, to the resonance being natural, you would, through the medium of auscultation, form your diagnosis by the state of the respiratory murmur. If the respiratory murmur was heard naturally over the whole of the chest, then you would arrive at the conclusion that the disease was situated merely in the larynx. It is often complicated, however, with bronchitis, and then you still find a good resonance, no dulness on percussion at any particular spot; but you have, to a greater or less extent over the large, and perhaps some of the small bronchial tubes, mucous, sonorous or sibilous rattle. This again is a condition which admits of being cured; and, therefore, you would make up your mind, under such a condition, that there was inflammation of the mucous tissue of the larynx, accompanied by inflammation of the mucous membrane of the bronchi."

These excellent remarks are followed by others of a similar character, respecting tuberculous deposits in the lungs, hepatization, excavations, &c. Chronic inflammation of the larynx produces, sometimes only a congestion of the lining membrane—sometimes thickening—hardening—roughness—tuberculation—ulceration. The ulceration may take place about the rima glottidis, or even in the epiglottis—about the chordæ vocales, ventricles—the angle in front of the thyroid cartilage. Mere softening of the mucous membrane is a degeneration which Dr. R. has seldom seen.

The treatment of this complaint must be, to a certain extent, antiphlogistic. In many instances, it will be necessary to bleed once, twice, or even oftener, from the arm, besides frequent leechings. Counter-irritation, by leeches, antimony, or, what Dr. R. prefers, croton oil, will be advantageous. Dr. R. warns the student against too much reliance on counter-irritation. Leeches must be again and again applied, while there is pain on pressure of the larynx. Dr. R. prefers the croton oil to the tartar-emetic, especially in females and delicate subjects. The pustules after antimony sometimes run much farther than is intended, and sloughs form, which leave unseemly eschars on parts exposed to view, as the throat and nape of the neck.

In respect to internal medicines, he thinks the most efficacious is mercury, given in such moderate doses as to raise ptyalism in the course of a few weeks, rather than in a few days. Nauseants are of some use, in conjunction with mercury, and the best is antimony. A narcotic is generally necessary, in combination with the mercury and antimony, to allay the irritation of coughing. Opium is best, if the constipation be not troublesome—if so, hyoscyamus.

Where there is reason to suspect thickening of the mucous membrane, iodine may be employed with the mercury. The recommendation of mercury in this disease is on the presumption, that there is no strong tendency to struma in the constitution. In our prognosis, we should always bear in mind that, sometimes, a sudden spasm of the glottis snaps the thread of life in a moment.

---

### Fatal Case of Chorea.

One of the most interesting and distressing cases that has ever occurred in our practice, long and extensive as this has been, was the following. A beautiful young lady (aged 16 years), the daughter of a distinguished military officer, lately came under our care. About five months before her death, and while in Dublin, she began to complain of some thickness of speech, as she termed it, and that her tongue seemed too large for her mouth. After some time, she observed that there were certain irregular motions in the tongue, which she could not restrain, and thus her speech was still farther embarrassed. The organ was frequently protruded beyond the lips. Gradually there came on irregular motions in various muscles of the body and limbs, till at length, when we first saw the patient, about three weeks before her death, there was scarcely a muscle in the whole frame that was free from involuntary motions in rotation. Very soon after, a great number of muscles were in action at the same time, without any order or regularity, so that she could not walk fiom one room to another without staggering, twisting, sideling, or even retrograding. The tongue now was actually too large for the mouth, and was frequently thrust out with great violence, which annoyed the unfortunate patient very much. She had been put on a course of purgatives, and the alvine excretions were always, even to the hour of her death, of the most horiible fetor and unnatural appearance.

The chorea at last rose in intensity to a kind of epilepsy, requiring two or three people to hold her in bed. She screamed violently, and sometimes did not sleep a wink for several nights in succession. Sedatives were employed in vain, and tonics made her worse. A few days before death, the eyes got bloodshot, the pupils dilated, and the pulse quick and strong. Leeches were applied to the temples, and one of them having penetrated a twig of the temporal artery, a great deal more blood was lost than was intended. All the symptoms were aggravated considerably. The spasms amounted almost to tetanus—the tongue was thrust out, and gnashed by the teeth, till it could scarcely be got within the mouth—the action of the heart was tumultuous and irregular—the whole frame, in fine, became completely exhausted—the spasms ceased, and she expired without a groan or a struggle!

Such a mysterious disease and unexpected catastrophe naturally excited a strong desire, on the part of the medical attendants (Dr. Thomson, Mr. Grant, Dr. ———, and Dr. Johnson) to investigate the cause; but great obstacles presented themselves. These, however, were overcome, and the body was carefully examined by Mr. H. J. Johnson, in presence of several medical gentlemen. Every part except the spine was inspected, but no trace of disease could be discovered in any organ of the body! There were found, however, two spiculæ of bone (one rising from the orbital plate of the frontal bone on one side, and the other from the sphenoidal bone on the other side), of remarkable sharpness, and one of them nearly a quarter of an inch in length, as sharp as a needle at the point, and as a knife at the sides. These were not discovered till after the brain itself had been dissected and removed, so that we could not examine whether or not there was any increase of vascularity or other appearance in the brain, corresponding with the positions of the bony spiculæ.

It will probably be doubted by many, whether such frightful phenomena can be fairly attributed to such small matters as two sharp points of bone on the internal surface of the skull. These may not have been the cause of the disease and death of our interesting patient; but they were the only causes that could be found. It was matter of regret, that we could not get permission to examine the spinal marrow; but it is very improbable that any thing would have there been discovered, to account for the terrible train of symptoms which hurried this young creature to an early grave!

When first seen, three weeks before death, although the choreal symptoms were then of unusual severity, yet it was prognosticated that this young lady would recover; but, in fact, the disorder rose from chorea into continued epileptic convulsions for more than a week before dissolution. The case is not a little remarkable, as well as melancholy.

---

### Extraction of a Brass Pin from the Female Bladder by a new Instrument.

The following case is contained in our northern contemporary, for July of the present year. It is extracted from an Italian Journal.* We confess that it is difficult to understand exactly the construction of the instrument, although the principle on which it was intended to act appears sufficiently clear. As the case may furnish an useful hint to surgeons, we transcribe it.

*Case.* Maria Ghizzi, aged 16, of irritable temperament, was long tormented by intense itching along the canal of the *urethra,* which she relieved by introducing into the canal a little fork of thick brass wire, and which she used, according to the custom of the country, to pin her hair. This instrument escaping, slipped into the *urethra;* and the attempt which she made to extract it by means of a large needle, with a buttoned end, thrust it completely into the bladder.

Dr. Bianchetti procured a *cannula* of polished steel, four inches long and three lines in diameter, with one extremity prolonged in the shape of a handle, and an iron rod, the upper extremity of which ended in a transverse bar for holding it, and the lower in a pincer, the toothed blades of which were separated by a spring, while the rod was furnished with a vice moveable by a screw.

The patient being placed on the edge of the bed, with the thighs bent on the belly and parted from each other, after dilating slightly the canal of the *urethra,* he introduced the *cannula,* and injected some warm water into the bladder. He then introduced through the

---

* Annali Universali di Medicina, Vol. LXXXV. p. 150.

*cannula* the metal rod already mentioned, and moved it in several directions within the bladder with the hope of meeting the foreign body. As soon as he felt that the little pin had fallen between the blades of the pincer, he introduced to a greater depth the *cannula,* the circular margin of which pressed the blades together and closed them. The operator then arranged the instrument in such a direction, that the foreign body occupied nearly the central portion of the cavity of the bladder—with the intention of protecting the walls of the organ from injury. Then passing along the vice of the metal rod, the screw which there rested against the upper end of the *cannula,* he, without any shock or uneasiness, drew the foreign body, which was held by the blades of the pincers, into the interior of the *cannula,* and thus extracted it without inconvenience. The patient returned immediately to her occupations.

It is extraordinary how foreign substances find their way into the urethra of the female. Mr. Thomas, of Leicester-place, was summoned to a lady by her husband, who gave a most incomprehensible account of her case. He found, on examination, that a silver toothpick had made its way into the bladder. We have heard of another case, in which a pin, used for braiding hair, insinuated itself into the same receptacle; and Sir B. Brodie removed several calculi from a lady, each of which had a piece of common hair for its nucleus. Industrious and unprofitable reading might, no doubt, multiply cases of this sort.

By the way, there appears to be a small degree of mystification about Dr. Bianchetti's manipulations. How did he contrive to get the hair pin into the canula? If it would go *into* that, we may be pretty sure that it would come out well enough through the urethra. In Mr. Thomas's case this was done, and a foreign substance that would go through a natural urethra, could be extracted through a dilated one. It may be said that, if a long instrument were caught crossways by the forceps, it could not be removed; but neither could it be got into a canula. In fact, unless we are utterly bothered by Dr. Bianchetti, his canula and his whole apparatus were much ado about nothing, and a simpler would answer equally well.

---

### Temporary Loss of the Memory of Words, after an Injury of the Head.

It is well known that injuries of the head have occasionally been followed by the temporary or permanent loss of language. Perhaps the most celebrated instance is that of the Welshman, admitted into St. Thomas's Hospital. He forgot the English which he had recently acquired, and remembered only the Welsh. M. Dupuytren has related a case, in which the patient named every object, and expressed every want, by the word pa pa. He could use no other. At Antwerp, after the siege of the citadel, we saw a man who had been wounded over the longitudinal sinus. He had only one or two words at his command. His intelligence was good, the power of moving the tongue, and, apparently, of combining the action of the pharyngeal muscles and those of articulation perfect; but his memory of all language, save a few words, was destroyed. Whether he ultimately recovered it, we do not know.

Dr. Inglis has related a case of gunshot wound of the head, in our Phrenological contemporary, which affords another illustration of the loss of language, from injury inflicted on the brain. We will give a slight sketch of the particulars, which are not, in a surgical point of view, uninteresting.

*Case.* A woman, æt. 33, was shot by a sheriff-officer on the 24th of Dec. 1835. The ball entered the cranium at the external orbital angle of the frontal bone. That night she had an epileptic fit, and she remained insensible till the 26th, on which day sensibility returned.

On the 29th, she was seen by Dr. Inglis and Mr. M'Keur, when she answered questions correctly, and her memory was quite unimpaired. She complained of some pain of the back part of the head, but especially of a dull, heavy, constant pain in the region of the wound. This was increased by some degree of vertigo, which followed from assuming the erect posture. A probe was introduced into the wound, and, after penetrating about an inch and a quarter, was stopped by a splinter of bone. On passing the probe a little to the right, or towards the median line, the bullet was distinctly felt, having penetrated both tables of the bone, imbedded deep in the rugged edge of the internal one. A sufficient incision being made, Dr. Inglis succeeded, by the application of some force, in extracting the bullet. It was flattened on one side and rugged, having the impression of the bone into which it had been impelled. Several splinters were removed, and also the one mentioned above of greater size, which was pressing upon a portion of the anterior lobe of the brain. The orbitar plate of the frontal bone was also considerably injured. The wound was dressed with adhesive straps, and lint, wetted with cold water, kept applied. The pulse was 72. A purgative was prescribed.

On the 30th, there was violent pain in the back of the head—pulse 80—bowels open. On the 31st, had slept little, and still complained of the pain in back part of the head. Appears drowsy, and answers questions incoherently. The wound discharges healthy pus. No stool since yesterday. Tongue white, but moist. On the 1st of January, Dr. I. found that she had been up and eating animal food—pulse 100—pyrexia—some degree of stupor, and talked incoherently when roused. On the 2d she was insensible unless roused, when she knew every body, and understood what was said. She seemed to have lost the memory of words, and could not express her wants. Pulse 68—pupils natural.

She continued in nearly the same state for the next two days, when, after excitement she become more comatose. Pulse 60. On the 6th, much discharge took place from the wound, and a small piece of bone came away. After this, she seems to have improved, for on the 9th, the power of speech was returning, and on the 11th, she could talk slowly and with hesitation. The wound was then greatly cicatrized, and the pulse had fallen to 48.

"All the symptoms have gone on improving down to the present date (Jan. 18). Still, however, she forgets some words; and, when talking about any thing, she repeats the same words several times before she can recollect others to express her ideas in succession; and often stops short in the middle of a sentence, telling the nurse to finish it for her."

In a postcript, we are informed that the memory of words is quite restored.

It will be observed that, in this case, the loss of the recollection of words did not follow hard upon the injury, but occurred in connexion with fever, and at a period which renders it probable that inflammatory action occasioned it. In this there is nothing to excite surprise; and we cannot wonder that this, as well as many other disturbances of the cerebral functions, should occur when its structure is interfered with. Such cases are sad stumbling-blocks to those who, not content with doubting the truth of the details of phrenology, dispute its general principles, and, surrounding themselves with a metaphysical fog of generalities, are unable to discern, or unwilling to admit, the most obvious facts.

There is a practical interest, as well as a physiological one, that waits upon this case. From a careful consideration and comparison of many instances of wound of the cranium and its contents, it has appeared that, on the whole, more mischief has followed the extraction of foreign substances from the brain, than from allowing them to continue undisturbed. Unfortunately, the case before us is so indistinctly worded, that it is impossible to decide whether the dura mater was torn or not. No mention is made of its laceration, yet it seems not unlikely to have happened, when splinters of bone and a bullet were lodged so deeply as they are said to have been. Of course this constitutes a very important feature of the case, whether we regard it in a physiological or a practical light, and it should not have been left in the uncertainty in which it is. The editors of our Phrenological contemporary would do well to exercise a strict surveillance on the cases and contributions of their correspondents, for some of them are jejune, if not inaccurate, and are calculated, perhaps, to bring discredit on phrenological observers.

---

Acute Pericarditis. Dr. Roots.

A case of this kind is published in the 4th Number of the St. Thomas's Hospital Reports, as a peg to hang some valuable clinical remarks, by Dr. Roots. The patient was a butcher, aged 30, admitted on the 17th Dec. who, after exposure to wet three weeks previously, was attacked with rheumatic pains about his shoulders and arms; but he did not discontinue his work. In a week, the pain settled in the region of the heart, accompanied by palpitation. He then was confined to the house—the joints of the lower extremities swelled, but did not change colour—motion was stiff and painful—surface of body covered with sweat—tongue coated and moist—thirst—confined bowels—pulse 98, and hard—respiration hurried. " A very distinct rubbing sound may be heard over the region of the heart." No medicines or means had been used. He was bled to eighteen ounces, and had five grains of calomel every six hours, after an aperient. The blood was buffed and cupped.

The next evening, the breathing was oppressed, and the cardiac pain increased. Again bled to 12 ounces.

Dec. 19th. The bowels are much relaxed. The pain in joints and region of the heart diminished. The calomel omitted, and half a grain of opium given every four hours. 20th. The calomel was again resumed every four hours. 21st. Much the same, but has cough, with frothy expectoration of mucus. The rubbing sound of heart continues. The bowels being much relaxed, the calomel was omitted.

" Dec. 22. Has slept but little during the night, and talked incoherently. The swelling of the joints has diminished considerably, but he is still unable to move them on account of pain. The breathing is still rather short and the cough increased. The rubbing sound is slightly lessened. The purging has diminished and the tongue is less coated. He is now in a profuse perspiration. Pulse 96. *Applic. Hirud.* xij. *reg. cord.* ℞. *Hydr.* c̄ *Cret.* gr. v. *Op.* gr. $\frac{1}{3}$ *4tis hor. Inf. Catech. Comp.* ℥j. *cum sing. dos pilul.*

Dec. 23. Has had a restless night and talked incoherently. The bowels continue purged. The tongue still covered with white fur, except a streak down its centre and its tip, which are reddish. The action of the heart is irregular, intermitting at almost every third beat, and he has rather more pain on pressing up the diaphragm and taking a deep inspiration at the same time. ℞. *Infus. Catech. C.* ℥ij. *sing. dos. pilul.*

Dec. 24. The bowels have only been relieved twice since the last report, and the tongue remains coated, its tip brown, and the central streak assuming the same character. The rubbing sound is less distinctly heard, but the pulse continues irregular, about 90, and will not bear much pressure. The cough has considerably increased, and the respiration is loud and sonorous. *C. C. ad* ℥iv. *reg. cord. Adhib. Cuc. Sicc. pectori et postea Emp. Lytt. amplum.*"

From this time he gradually but slowly recovered, and was discharged on the 9th Feb.

Dr. Roots remarks that this case offers an answer to those, who say that " bleeding in acute rheumatism gives rise to inflammation of the pericardium." We confess that we never heard such an observation made. We have often, indeed, said, and we still believe, that acute rheumatism will not bear bleeding in the same way, and to the same amount, as common inflammation, say of the pleura; nor will it be cured by bleeding alone. We have seen many instances, also, where large sanguineous depletions appeared to produce metastasis, especially when syncope was induced, and the joints suddenly grew pale and shrunk. It is to be recollected that the pain in the region of the heart, in the above case, commenced *before* the joints became affected, so that, in fact, we had here pericarditis before the existence of *acute* rheumatism, and, consequently, the venæsection was necessary and proper for that part of the complaint alone. We see, in this case, no proof that there was metastasis from the joints to the pericardium. The pericarditis was at least coeval with, if not antecedent to, the acute rheumatism.

We agree with Dr. Roots, that the *bruit de soufflet* is not essential as a symptom in pericarditis.

"I merely call your attention to this fact, because in many instances the stethoscope fails to give us any positive indication as regards the existence of inflammatory action going on in the pericardium. You may have, and more especially in the commencement, acute inflammation of the pericardium, without any indication by auscultation to warn you of its existence. It is true that if, in conjunction with the general symptoms of pericarditis, you find a bellows-sound, that would be some corroboration, because you do often meet with a bellows-sound, but still it would only be corroborative of other symptoms."

Pain in the region (dull or acute) and dyspnœa are the most common symptoms in pericarditis. The pain is generally increased by inspiration, and often extends backwards to the shoulders and scapulæ, or to the left arm. There is anxiety of countenance, and inability to lie on the left side. The pulse varies much. If effusion to any extent takes place, it becomes irregular. In respect to the bellows-sound, Dr. R. thinks it chiefly occurs when the lining membrane of the heart itself is inflamed, in addition to the pericarditis.

The rubbing sound, like the creaking of new leather, Dr. R. is inclined to attribute to the effusion of lymph on the investing surface or surfaces. It appears to him very doubtful that it is owing to incipient adhesion.

"With respect to the treatment that was pursued in this man's case, you will remember he was bled twice from the arm, was cupped, and was leeched repeatedly: and though the rheumatic inflammation was considerably diminished, as the effect of the bleeding, yet still the heart never lost, to any considerable extent, its rubbing sound, until his system was put under the influence of mercury. As soon as his mouth became sore, Mr. Jones, who closely watched the case, observed that the rubbing sound diminished. This would probably have been the case sooner, but in consequence of the highly irritable condition of the mucous membrane of his bowels, we were obliged to suspend for some days the use of the mercury, and thus some considerable time was necessarily lost before his bowels resumed such a condition as to bear it; but so soon as his mouth became sore, the rubbing sound diminished. He was kept for some time under the influence of the mercury, and the sound entirely ceased. The treatment adopted was that which is proper for pericarditis generally. You will not find it perhaps always necessary to take blood from the arm in those cases which come into the hospital, because they do not often come here in the early stage. In this case it was absolutely necessary to take blood from the general system, but afterwards local depletion was principally depended upon."

Dr. R. observes that his success in the treatment of pericarditis is much greater now than it formerly was. The reason, he thinks, is, that he now keeps his patients longer in hospital, and suspects that he had often turned them out too soon—that is *apparently*, but not really cured.

"Having had an opportunity of seeing two or three such cases return to me with renewed inflammatory action, I have since that time adopted a different plan, viz. as long as there was this increased activity about the heart, as long as there was any thing like a bellows-sound, provided it was not of old standing, the result of former attacks, I have invariably thought it right to keep my patient for a greater length of time under the influence of mercury, and frequently from time to time to apply either leeches over the region of the heart or cupping-glasses, so long as there was the slightest proof of insidious inflammation still existing; the consequence is, I may say in the three last cases the patients perfectly recovered."

When the heart remains irritable, after the inflammatory symptoms are subdued, he gives small doses of colchicum, either alone or in combination with digitalis — sometimes combined with henbane or opium.

---

### The Statistics of Hernia.

Our northern contemporary for July contains a paper by Dr. Knox upon this subject. It displays, perhaps, from the nature of the subject, a considerable number of negatives: and the statistics of hernia remind us of the chapter in Fielding, entitled, "Conclusions, where nothing is concluded." On perusing the article we may exclaim with Harold:—

> Well has he said—Athena's wisest son—
> All that we know is—nothing can be known.

We leave the greater number of the uncertainties to shift for themselves, and notice only some of the data which Dr. Knox has brought before us.

1. The question has been frequently mooted—What is the ratio of ruptured persons to the entire population? One author has affirmed one thing, another author has affirmed another thing, and, as from the very nature of the case there can be no positive available data, we may leave the matter without further ceremony in abeyance. It has been attempted to arrive at a decision by ascertaining the proportion of the ruptured in a certain number of recruits. But those only present themselves for recruits who are comparatively healthy and strong; and unless a most unsparing conscription were resorted to, we should form very limited conclusions from such premises. It appears that Dr. Knox has calculated from the results of a three years' conscription in France, that about $1\frac{1}{3}$ per cent. of the whole male population have hernia. This is, probably, not very far from the mark.

The memoirs of the French Academy of Surgery furnish the following facts.

| | |
|---|---|
| Received into the Salpetriere .......... | 7027 women, |
| Of these were ruptured, | 220 do. |
| Received into the Bicetre | 3800 men, |
| Ruptured............ | 212 do. |
| Received into the Hôtel des Invalides.. ... | 2600 men, |
| Ruptured ............ | 155 do. |
| Received into the Hôpital de la Pitié.... | 1037 young men, |
| Ruptured ............ | 21 do. |

Thus amongst the men we have about 1 to 19 ruptured; whilst of the women we have about 1 in 32 affected.*

2. Are particular classes more liable to be affected with hernia than others?

It is generally believed that the labouring classes *are* more affected than those whose circumstances withdraw them from the necessity of corporeal exertions. Dr. Knox demands the positive data. But it appears to us that this is rather unreasonable; for there are no possible means of arriving at the

---

* It appears from the Globe Newspaper of April 13th, that "The Rupture Society of London held a meeting last week. This society has been instituted since 1805. From the records kept by them, it appears one in fifteen at least (of both sexes) are affected with this complaint, and among such as are exposed to great bodily exertion, the average is one in eight or nine.

31,400 cases have been relieved by this society since 1805." This is probably a little *de trop*, and the Rupture Society have perhaps been not unwilling to receive statements, favorable to the sense they entertain of their own beneficial exertions for humanity.

Dr. Knox has a whimsical notion; he supposes that there are whole classes of society nearly exempt from hernia—the *very rich*, and the *mendicant by trade*. The statistics of both these classes are rather difficult of attainment.

numbers of cases of rupture in the better classes, the complaint being one that they endeavour to conceal. When we see that bodily exertions are constantly producing hernia; when we reflect that the lower classes must, ex necessitate, make such; and when we observe, as every surgeon must do in practice, that the cases which he himself witnesses are proportionally more numerous in the class in question than in any other, it is difficult to avoid arriving at the conclusion, although not demonstrably confirmed, that the general belief, drawn from general experience, is correct. Demonstration is an excellent thing; but where the nature of the case does not permit it, we are forced to form our opinions upon evidence less satisfactory. Of course the farther that evidence recedes from the demonstrative kind, the more suspicious we must be in its reception, and the less confident in its absolute accuracy.

3. Is rupture more prevalent in males than in females—on the right side of the body than on the left? It is commonly believed to be so. Here again general experience has pronounced an affirmation, and general experience, on mere matter of fact, is seldom very wrong. We have certainly seen hernia much more frequently in males than in females—it is more frequently the subject of hospital operations in the former than in the latter—and we would almost go so far as to assert, that for one hernia on the left side we have found two upon the right. But on these points there are *some* data.

In the first place, to revert to the memoirs of the French Academy of Surgery. Out of 7027 women in the Saltpetriere we find only 220 ruptured; while out of 7437 men we find 388 ruptured.

The following additional facts are quoted by our author.

" From a Report of the City of London Truss Society for the year 1814, it appears, that of 7,599 persons to whom that institution had afforded relief from the period of its first establishment, 6,458 were males, and 1,141 females.

The new rupture society had relieved 3,505 males and 565 females, but I cannot consider this as affording any data for establishing the relative frequency of hernia in the sexes; it merely shews that more males apply for relief than females, a circumstance every one must know very likely to happen in hernia.

In respect to the ratio of occurrence on the right or left side, the following table exhibits all I am acquainted with on the subject.

*Tabular view of the comparative frequency of inguinal and crural hernia on the right and left sides.*

| | | | | | |
|---|---|---|---|---|---|
| Of 7,599 cases of hernia there were, of | | | | | |
| Inguinal Hernia, | Males, | 2567 | Right side. | 1469 | Left side. |
| do. | Females, | 20 | do. | 14 | do. |
| Femoral Hernia, | Females, | 264 | Right side. | 246 | Left side. |
| do. | Males, | 47 | do. | 38 | do. |
| Ratio, according to the Report of the New Rupture Society. | | | | | |
| Inguinal Hernia, | Male, | 1563 | Right side. | 927 | Left side. |
| do. | Female, | 51 | do. | 34 | do. |
| Femoral Hernia, | Female, | 139 | Right side. | 93 | Left side. |
| do. | Male, | 19 | do. | 11 | do." |

The numbers of hernia on the left side, not stated in the table, are, of course, the remainder, after deducting the numbers on the right side from the total amount.

It is generally believed that the greater frequency of hernia on the right side, arises from the more severe exertions made with that side of the body. Dr. Knox, however, appears to believe that it depends on a greater size of natural apertures, &c. on that side. The following are his principal grounds for this opinion.

" There is an anatomical structure which when present seems to me positively to predispose greatly to hernia, I mean great width of pelvis, and consequently increase in measurements of this cavity whether male or female. I remember examining the pelvis in a male who had double ruptures on each side, that is an inguinal and ventro-inguinal and femoral hernia on both sides; the size of the pelvis was much greater than is usually met with in the male, in fact it approached that of the female in all its dimensions, but before it was practicable to measure it when stripped of its soft parts, and then compare it with others, the specimen was lost. My impression was, that it nearly equalled in all its dimensions a full-grown female pelvis. Moreover, in two cases of strangulated crural hernia which I had occasion to see in the male, the pelvis in both was much larger than usual, in fact it approached the female in its dimensions. I regret exceedingly that I had no opportunity of measuring the pelvis in these cases; but from what I have seen I feel disposed to think, that an unusual size of pelvis in the male is a frequent predisposing cause of inguinal hernia, and when extreme, even of crural hernia, which is not so uncommon in the male as some have supposed. The hernia had not been detected during life in the two cases I speak of, and they died in consequence of strangulation."

"I next attempted to ascertain, by measurement, if the right side of the pelvis has a greater development than the left, and what ratio; but after several attempts I found it extremely difficult to prove by the compasses to how much the difference amounted. It was obvious that in the specimens examined, by far the greater number shewed a greater width of pelvis on the right side than on the left. I speak of the measurement from the *symphysis pubis* to the base of the anterior and inferior spinous process of the *ilium;* whilst in a few the left side was rather the wider, and in others the parts were quite symmetrical. I found the same discrepancies in regard to the *foramen obturatorium.* But there is a form of male pelvis, frequent enough, and which convinces me that there is a tendency to a greater development of the pelvis on the right side. The form of which I speak is one where the left *ramus* of the *pubis* descends almost straight to meet the corresponding *ramus* of the *ischion,* whilst on the right side the ramus arches widely out, producing a singular want of symmetry in the arch of the *pubes.* The same want of symmetry happens not unfrequently with the clavicles, which may be the reason why, generally speaking, the surgeon finds more room above the right clavicle for the ligature of the corresponding subclavian artery than above the left, which not unfrequently is smaller, shorter, and less arched. I have subjoined outlines of the three extreme forms which the arch of the pubes assumes, fig. 1st, (Plate II.) shewing the perfectly acute or pointed arch, a form, I presume, which induced M. Meckel to call it the 'angle of the pubes' in the male; 2d, The rounded arch of the well-formed female pelvis; 3d, The symmetrical or hermaphrodite pelvis, in which the two sides are formed on different plans,—the right almost uniformly presenting a much greater capacity than the left side, but which difference, for obvious reasons, can scarcely be ascertained by actual measurement. This form of pelvis is by no means unfrequent in the male."

We have taken the liberty of running together these two quotations, although they are disjoined in the paper. They display our author's ideas upon the subject. It appears to us that an increase of width on one side of the pelvis, so indefinite as not to be susceptible of determination by measurement, will with difficulty account for the much greater frequency of hernia on that side. It must be remembered that inguinal hernia is the ordinary form in the male, and it is extremely doubtful if *such* a cause would sufficiently explain that. For our own parts, we incline to the ordinary explanation of the fact

What is the proportion of the different varieties of hernia to each other? On this point we do not appear to have very extensive data. Dr. Knox copies the following statements from M. Cloquet. This surgeon dissected 457 cases of hernia, and amongst these there were only 3 in men, "umbilicales et de la ligne blanche," in other words, of 457 cases of hernia, selected from many thousand dead, M. Cloquet met with three only in man, "umbilical and ventral." Of 7599 cases relieved by the London Truss Society, there were only 44 ventral. These statements are in reference to a declaration on the part of Mr. Marshall, who, in reference to the disabilities of recruits from hernia, has observed that, out of 82 cases of hernia, he found 50 to be ventral and umbilical—a monstrous discrepancy from the observations of all other surgeons.

---

### Tight Lacing.

In a clever little work recently published by an intelligent surgeon—Mr. Coulson, there is a chapter dedicated to tight lacing—chiefly taken from a work by Professor Soemmering, on "the Effects of Compression of the Waist by the *Use (?)* of Corsets."* Every one knows that the thorax is, naturally, of a conical form, the apex being uppermost. Now the shape of a lady's (or, indeed, of a gentleman's) stays is quite the reverse—their narrowest part being below—that is to say, the smallest diameter of the stays encompasses the largest part of the chest, and *vice versâ*. Further, the stays are hard or stiff below, where the abdomen is or ought to be moveable.

"The use of the stays, when they have the least effect on the chest, produces compression of the soft parts below, and throws up the viscera of the abdomen towards the chest.

Not only will the moveable false ribs be pushed upwards, and close together, and the space between them diminished, but they will be so pressed, that those of the right side will be brought nearer to the left, not only at their anterior extremities (the last, perhaps, excepted on account of its shortness), but also at their extremities towards the spine. In consequence, the inclination of the false ribs generally must increase, and their cartilages be more bent; for the cartilaginous parts yield most readily, and the bony parts, on account of their elasticity, yield also a little.

If the compression be carried further, the lower true ribs will be carried upwards towards one another; the right will be carried towards the left, the sternum will ascend, and when the pressure is increased, the sternal extremities of the lower true ribs will necessarily be brought nearer to the spine, and the diameter of the chest, from before to behind, be diminished.

Whilst this is going on with the ribs, the bodies of the vertebræ are somewhat raised, their spinous processes gradually become more oblique, and pressed on one another, and at last the spine becomes bent.

Superiorly, the thorax naturally becomes smaller. The fifth and sixth ribs do not further suffer from the immediate pressure of the stays, but commonly form more or less of a circle round the chest. In the remaining upper ribs, the contrary, to a certain degree, is the case: the ribs are pressed from one another by the internal viscera; their interspaces are greater; the right is somewhat separated from the left; and their sternal stand off from their spinal extremities.

To the act of breathing, the first, second, third, and at the utmost the fourth ribs, contribute: it even appears as if they were more moveable.

To this space are the breasts, with the surrounding parts, pushed upwards, and such persons appear to have larger breasts, but some part of these organs usually suffers from the pressure.

The shoulder-blades are sometimes brought closer to one another behind; and their under part is pressed towards the spine; the back loses its fine rounding, and the arm is impeded in its free motion. Hence, when a tight-laced person, while sitting, reaches over, she must move the whole upper part of the body on the hips."

If all these changes take place exter-

* Ueber die Wirkungen der Schnurbruste.

nally, what must the internal organs suffer? The lower portions of lung are compressed—the circulation is impeded—the diaphragm is pushed up forcibly, and embarrassed in all its motions. The viscera of the abdomen suffer. The stomach is compressed, and bad digestion follows. The duodenum is pushed upwards unnaturally, and the function of the liver is impeded. The rectum, uterus, and bladder are forced lower down than is natural. From two measurements, Soemmering found that, "in a fine girl, the circumference of the *head* was twenty-two Paris inches; while the circumference of the waist, with the stays on, was 21 inches and a mere fraction." In another girl, the circumference of the head was 18 inches, and that of the laced body was 15 inches! In this last, the circumference of chest, at the arm-pits, was 39 inches. The body was, in this young lady, three inches less in circumference than the head!

Soemmering has collected from various authors, and with true German industry, a catalogue of the diseases or disorders produced by tight lacing—and they amount to the frightful number of—NINETY-SIX! Many of them, too, are amongst the most formidable and fatal maladies to which flesh is heir! We need only glance at a very few of these, viz. carotidean aneurism, cancer, asthma, hæmoptysis, pulmonary abscess, consumption, hydrothorax, scirrhus of the pylorus, dysentery, jaundice, cancer of the womb, &c. &c. Amongst numerous evils enumerated by the Germans, as attributable to tight lacing, is "ugly children." It is also to be borne in mind, that habit renders the tight stays necessary; for ladies, after 15 or 20 years, are so dependent on them, that they cannot keep themselves erect without them.

The injurious effects of tight lacing are hardly exaggerated by Soemmering or Mr. Coulson; for the abdomen and thorax are so much compressed by the stays, that the ribs cannot rise nor the diaphragm descend at each inspiration. Neither digestion nor assimilation can, therefore, be properly carried on, and many of the corporeal functions must necessarily languish. The lower lobes of the lungs being compressed, there must naturally be a greater onus thrown on the upper lobes, especially in singing, dancing, &c. And this, we apprehend, may be one cause, especially among the upper classes of females, why we generally find the lower lobes hepatized, in those who die of consumption. This same tight lacing is considered essential to a fine figure, and yet we never could discover any marks of stays in the statues of the Medicean Venus or Belvidere Apollo. Whether the modern girdle possesses any of those attractions attributed to the cestus of Venus, we will not pretend to determine: but we venture to aver that the Cyprian Goddess was not in the habit of drawing her zone so tight as the modern fair ones are, else the sculptor would have recorded the cincture in Parian marble.

Tight lacing is not confined to the female sex. Let any one look around him in the streets, the theatres, the ball-room, &c. and he will acknowledge that the beaux are nearly as tightly girt as the belles. The mania pervades the dandy creation, from the Neva to the Hellespont. The HUN and the CROAT have their upper regions as nearly severed from their netherlands, as the Gaul and the Italian. John Bull has caught the phrenzy, though his well-stuffed paunch makes a desperate resistance to the girdlo-mania of the Continental fop.

The public are indebted to Mr. Coulson for bringing this subject prominently forward at the present time.

---

### POISONING BY CANTHARIDES.

In the Jamaica Physical Journal, for May, 1835, Mr. Maxwell, surgeon to the Annotto Bay Marine Hospital, has related the cases of three negroes, accidentally poisoned by cantharides. They did not die, and we shall greatly abridge the details.

It appears that six drachms of finely-powdered cantharides had been macerated for eight hours in a bottle of rum.

The contents of the bottle were divided pretty equally between three negroes, though, as it afterwards turned out, three drachms of the powder remained untouched at the bottom of the bottle. This was at 8, p.m. of the 16th. One of the negroes had previously eaten his supper—the other two supped immediately afterwards.

In a short time they complained of sharp lancinating pain of stomach, burning sensation of the throat, with great nausea, which increased, and in a couple of hours they were seized with violent retching; the contents of their stomachs were mixed with blood, mucus, and froth.

The kernels of six antidotes *(Feuiella Cordifolia)* were boiled in a pint of water, and given to each by the black dispenser, which acted as an emetic.

Early next morning Mr. Marshall saw them. "They are all complaining," he says, "of excruciating burning pain in the region of the stomach; surface covered with cold clammy sweat, sense of constant burning of the throat, most intense at the top of the œsophagus, and descending along that passage to the stomach. There is great difficulty in swallowing, and to use the significant expression of one of them, his throat is on fire. There is great depression of the vital powers*—they are cessantly moaning, and consider death to be inevitable; pulse accelerated about twenty beats in a minute, and rather weak. The breathing is not difficult, nor do they complain of any irritation of the urinary organs; no pain or tension of the abdomen: the bowels have not acted since they took the poison. The vomiting recurs at intervals, and they eject frothy mucus in great quantity, tinged with a vermilion colour. They are regularly salivated, spitting incessantly a mixture of mucus and saliva of a pink colour. The throat of each was examined, and found to be swollen; had an erysipelatous blush of inflammation, with turgid veins running across the fauces. To be perfectly satisfied that every particle of the powder was removed, I administered twenty grains of the sulph. of zinc to each, which acted instantaneously: they drank copiously of warm water, and after their stomachs were settled, three ounces of fine olive oil was given to each, which they retained, Enemata of the infusion of ochro *(Hibiscus esculentus)*, with castor-oil, ordered to be exhibited frequently, and the infusion to be drunk ad libitum."

At 1, p.m. they had had no recurrence of vomiting, and one had had a copious stool, unmixed with blood. *Two ounces of castor-oil for each.* This operated on all without producing blood.

*Evening.* They spit mucus and saliva tinged with blood incessantly. Constant burning pain in the stomach—much difficulty of swallowing. Pulse of two 100—of the other 80. Blooded to eighteen ounces. Applic. emplast. lyttæ regioni gastrici. The enemata were directed to be given occasionally, and the ochro tea to be used as a common drink.

After the bleeding, the gastric symptoms in all were moderated, but strangury became developed. The throat, and even gums, displayed in all more or less of inflammation, and even sloughing, and a copious discharge of saliva ensued. We think it is useless presenting any farther details. In two or three weeks, all the patients had perfectly recovered.

It is evident that the cantharides acted more as a local irritant, than as a poison to the general system. The inflammation of the mucous membrane of the stomach, œsophagus, pharynx, and even mouth, was clearly the result of the application of the fly. Its injurious influence appears to be principally exerted on the mucous and cutaneous tissue. As, when absorbed, it is discharged by the urinary apparatus, we cannot wonder that it should occasion strangury. It is singular that

* "Cantharides in powder, taken into the stomach, acted as a corrosive, producing vomiting, the discharge of much bloody mucus, depression of the vital power, and death.

The stomach is always violently inflamed, and the bladder sometimes."—*Orfila, Traité de Poisons.*

these negroes only suffered from this symptom after the venæsection. Was this owing to the additional activity of absorption produced by that measure; or was it due to the blisters which, at the same time, were applied to the skin? We confess that we should be rather averse to their employment on a similar occasion.

---

### M. Breschet's Operation for Varicocele.

Our surgical readers are, no doubt, well aware, that a great number of plans have been devised, and that not a few have been actually employed for the cure of varicocele. The veins have been tied, divided, made to slough by the application of caustic, subjected to pressure, and the result has hitherto been that surgeons, after all, have arrived at the conclusion, that the best way of treating the complaint is not to meddle with the veins at all.

But so long ago as December, 1834, a correspondent of our contemporary, the Medical Gazette, discovered that the apprehensions of surgeons were a bugbear, and announced that the honour of discovering "a radical treatment" for varicocele, had been reserved for M. Breschet. "This distinguished surgeon," wrote our contemporary's friend, "has, in fact, devised a plan, simple as it is ingenious, by which the most inveterate scrotal and spermatic varices are completely and expeditiously removable." We intended, at the time, taking notice of the subject, but it escaped our recollection, and has recurred to us in consequence of seeing it recorded in the American Journal for November last. Before we make any further observations, we will extract sufficient to enable our readers to perceive what M. Breschet's method actually is.

The principle on which it is founded is to obliterate the affected vein by compression.

"The compression was at first produced by small iron pincers, the pressure of which always equal, and resulted from the simple elasticity of the branches of the instrument. But the difficulty of using these pincers, their occasionally too great pressure, and the impossibility of padding their metallic surfaces, induced M. Breschet to use in their stead others so formed, as to permit the part destined to come in contact with the scrotum to be covered with soft linen, or leather, and the pressure to be graduated at will, by means of screws.

The instruments were at first applied on the veins of the scrotum, at the extremity of two of the most voluminous of them; care being taken to leave no considerable anastomosis between the two points compressed. Their presence determined slight local pain, and the trifling inflammation which followed, was combated by emollients, resolutives, and repose. On a second application, the pain was much less considerable; the graduated compression caused a thinning of the skin, and, bringing the two opposed portions of that membrane into contact, produced a dry eschar, solid, thin, and transparent, resembling parchment. Its detachment was followed by an ulceration of much less extent, and less painful than in the former instance. These ulcers were perfectly cicatrized in a few days; they produced no hæmorrhage. The vein remained full of clotted blood; this blood was gradually absorbed; no inflammation took place; and subsequently all traces of the existence of the vessels had disappeared. The different veins of the scrotum were successively treated in the same manner.

This success was, however, incomplete. The spermatic veins still remained. The application of the instrument to them was not so easily effected. They were surrounded by a thick skin containing fat, and the vas deferens was in their immediate vicinity. The small size of the nerves and arteries prevented their being drawn aside. The accidents to be apprehended were pain, violent inflammation, formation of a deep eschar, and denudation of the cord. For this new application the instruments were modified, so as to offer a larger surface to the part affected, and to present a curve, which should prevent the pressure of the fold of the skin. The vas deferens was excluded. The pain on the application was considerable, but removed by local means. The instruments were kept on seven days; one placed as near as possible to the external ring, the other towards the inferior part of the cord. They determined an inflammatory swelling of the parts comprised between the two pincers, and produced a superficial eschar of the part on which they immediately acted, combined with an adhesion of the two opposed cutaneous surfaces. The consequent ulcerations were cicatrized in less than fifteen days; before which time the cord had lost its knotty feel. There, however, still existed, at the cauda of the epididymis, a mass of vessels extending as far as the place of the last compression. Two other pincers were applied; one immediately before the testis, which was drawn backwards, the other two inches higher up. They were kept on the same number of days as the preceding, and followed by some pain, inflammatory swelling, and eschars of the integuments.

It deserves remark, that the inflammatory symptoms, after this last operation, were more violent than before, and less promptly removed. The instrument placed above the testicle produced a perforation of the skin, and a solution of continuity resembling that produced by a seton. The inflammation and swelling were considerable; an abundant oozing of a sero-purulent matter existed during fifteen days, from the ulcer. These accidents were got under by emollient cataplasms and repose, and at the same time the varicose mass which had existed at the bottom of the scrotum, was converted into a small tumour, in which no trace of vessels was discoverable. The disappearance of the swelling soon permitted an examination of the testicle, which had been long lost amid the vast fasciculi of vessels. Its nutrition was found to be unimpaired, and its volume equal to that of the opposite side. The cure was perfect at the end of November."

The gentleman informs us that M. Breschet has operated in *numerous cases*, both in public and private practice, with perfect success.

We have no hesitation in stating explicitly and boldly, that, in our opinion, this is essentially an objectionable operation; and the following are our reasons for this dogmatic assertion.

In the first place, it is attended with danger. Inflammation is excited in the spermatic and the scrotal veins, and venous inflammation, however occasioned, will at times prove fatal. We believe that Sir E. Home lost a patient in whom he tied a spermatic vein, and M. Delpech, if our memory serves us, lost a patient also, after the application of caustic. Surgeons have almost universally abandoned operations on the saphena vein, because dangerous or fatal phlebitis every now and then ensued. It is idle to protest that compression is not so injurious as division by the knife, or the employment of the ligature. When we read the case of M. Breschet, and when we reflect on what must happen, it is obvious that inflammation, more or less severe, of the spermatic veins, must be produced, and it is that inflammation, not the exciting cause of it, which is hazardous. Our first objection, then, is the danger of the operation.

In the second place, the operation is not likely to succeed. The anastomoses between the spermatic veins are so numerous, and they constitute in themselves such a venous plexus, that it can be of little service obliterating the trunk of one or two, or indeed of any partial number. But if we obliterate all the veins, how is the blood to get back from the testis? Either we must leave veins to carry on the circulation, or we must tie the spermatic arteries also. The operation has been founded on a supposed analogy between varices of the veins of an extremity and varicocele. But that analogy is in some measure a false one; for, in the former case, the venous system of a limb is only partially affected; while in the latter it is almost the entire venous system of the testicle that is concerned. We are confident that the operation, painful and hazardous as it must be, presents little chance of permanent success. If only some veins are obliterated, the others will increase, and if all the accessible veins are choked up, fresh varicose veins will gradually form. The blood goes to the testis still, and it is ridiculous to suppose, that the way to favour its return is by opposing fresh difficulties. This is driving out devils by Beelzebub, the prince of them.

In the third place, let us pause and inquire what is this disease, for which a tedious, painful, dangerous, and, after all, a probably inefficient operation is proposed. Why it is so common, that the majority of men suffer in some degree or other from it, and it is so slight, that it very rarely amounts to more than an inconvenience. At the worst, it only occasions wasting of one testis, as it is extremely uncommon for the right to be affected; this cannot be regarded as a very serious calamity. Whether serious, however, or not, it is rather probable that the operation would tend to increase it, if it exerted any good effect at all. Yet, with all these facts staring us in the face—with the knowledge that varicocele is very seldom more than a source of some annoyance, requiring a bag truss and the bidet, we are told that M. Breschet has operated in "*numerous cases*," public and private. What conclusion can we draw, but that M. Breschet has subjected *numerous* individuals to an operation, to pain, distress, and hazard, without any material necessity, and, we will add, without much ultimate material benefit.

---

### Irish Unanimity; or the Blessings of Medical Corporations.

We extract the following passage from a little pamphlet on Medical Reform, by Mr. Andrew Ellis, one of the surgeons to the Charitable Infirmary, Jervis-st. Dublin.

"The selfish and coercive system, which pervades the three medical corporations in Ireland, appears to me to be an intolerable grievance, of which students have much reason to complain. At Trinity College, no certificates, but those granted by University professors, will be recognized; on the other hand, the College of Surgeons, in the true spirit of retaliation, will neither receive the certificates issued by the professors of any University, which will not recognise those given by the lecturers in their school; nor those granted for attendance on the lectures delivered in the school connected with the Apothecaries' Hall; and again, in return, the examiners at the hall will not acknowledge the certificates of the lecturers in the School of the College of Surgeons. Are we still to be told, that the continuance of this system of reciprocal retaliation and coercion is necessary for the advancement of science, and the preservation of the public health?"

"An Irish Apothecary cannot, as the law at present stands, open a shop either in England or Wales, unless he shall have first complied with the regulations, and submitted to another examination, before the London Court of Examiners. In like manner, the English Apothecary is prevented practising his profession in Ireland, until he shall have conformed to the regulations, and submitted to a second examination at the Hall in Dublin. The absurdity of the present state of the law on this subject, becomes still more palpable, when it is recollected, that both the London and Dublin apothecaries may, if they please, go and open shops, and practise, without restraint or annoyance, in Scotland!"

---

### Lithotomy and Lithotrity.

When any discovery is made, there are always plenty of half-witted enthusiastic people, who expect from it a sort of millennium, and who, as they are little better than fools themselves, are liberal in applying the term to those sober folks who exercise the privilege of thinking. But when a little time has elapsed, and actual experience has shewn the futility of extravagant anticipations, when the real merits and demerits of the plan have been made apparent by positive results, then these same people are apt to turn round, and vent the disappointment of their own unreasonable fancies on the measure that occasioned them. As love more easily runs into hate, than into dispassionate judgment, so enthusiasm, based on ignorance, exhibits its equally violent extremes.

We remember well, that when lithotrity was introduced, lithotrity with the objectionable three-branched instrument and drill, numerous correspondents in the Journals were loud in their denunciations of those English surgeons, who did not at once throw away the staff, and gorget, and knife, and assume the drill-bow and the rest of the lithotritic apparatus. Yet it is but the other day that a writer in the Lancet denounced a gentleman, eminent for his surgical attainments, because he had recommended a patient to be operated on by Baron Heurteloup. That patient died, and the writer demanded a list of the victims to the new process. Such inquiries as this, and such advocates or opponents of practical improvements, we may safely consign to their natural obscurity, and proceed to estimate, in a more candid and more rational manner, the advantages or disadvantages of lithotrity.

We have no intention of entering in an elaborate manner on a comparison between lithotrity and lithotomy. Such a comparison can only be accurately drawn from facts—at least they must form a considerable portion of its basis. But, from the very nature of lithotrity, we are kept very much in the dark with respect to it. We hear much of its success, and little of its failures. As it has been hitherto chiefly practised by those who have devoted themselves exclusively to its performance, they have naturally been chary of publishing what might alarm the world. Yet something has transpired—a good deal is known—and there are data for arriving at an approach to truth in our conclusions.

We shall first direct attention to some discussions which have taken place in the French Academy, and to some letters which have been published in Paris by MM. Civiale, Velpeau, and others, and we shall then subjoin a very few remarks of our own. For what occurred in the French Academy, we are indebted to an article in our contemporary, the American Journal of the Medical Sciences, from which we shall draw the succeeding statements.

It appears that, in France, that is in Paris, the advocates of lithotrity and of lithotomy constitute two positive, if not violent factions. Throwing off the calm and philosophic spirit which should characterize the pursuit of scientific truth, they have disputed with all the acrimony of political partisans, and exaggerated, as such brawlers do, not only conclusions, but premises. Four successive sessions of the Royal Academy witnessed their furious discussions, and the report by which they were said to be concluded, but will be really prolonged, was adopted amid "incredible tumult"—protests against the manner of voting—and demands that the question should be put again in the succeeding session.

1. The discussions in the Academy were, in the first instance, occasioned by a memoir of M. Leroi d'Etiolles, on the practicability or utility of lithotrity in children. Our readers are no doubt aware, that it has been generally agreed that the operation is not applicable to very young persons, on account of the small size of the urethra. M. Leroi, however, has presented the history of five cases in which lithotrity was attempted or performed, and applied the results to the solution of the question. The following is the abstract of the memoir contained in our contemporary.

"1st case—a child of four years. *Stone* nearly an inch in diameter. It was very fragile, but required six operations for its removal. *Accidents.* Two fragments, at two different times, became engaged in the urethra, occasioning 'a great deal of suffering.' *Result.* Cure. 2d case—age five years. *Stone* about the same size, destroyed by five operations. *Accidents.* None mentioned. *Result.* Cure. 3d case—age not noted. *Stone* about the size of a filbert, partially engaged in the urethra; seized and broken with forceps with three branches. *Accidents.* On the morning following the first operation, a fragment passed the prostate gland, became arrested in the urethra, and caused 'violent pain.' Two days afterwards, another fragment was arrested in the same manner and produced the same consequences. It was repulsed with 'infinite difficulty,' nor was it broken with less. *Result.* The child ceased to suffer, but as the patient was unmanageable, the cure could not be rendered certain by sounding. 4th case —age four years. *Stone* very small, and broken twice with great ease, the child being admirably manageable. Four operations are noticed in the report. *Accidents.* At the third operation, a portion of the forceps remained in the bladder, *a fact only known to the operator;* this was removed a few days afterwards by means of another forceps. A fragment of the stone was engaged at the same time in the urethra, and could not be pushed back immediately. *Result.* M. Dupuytren took charge of the patient, pushed back the fragment, and performed the bilateral operation. The patient was thus cured. 5th case—age three years. *Stone* three or four lines in diameter. *Accidents.* None. *Result.* Cure after two operations."

M. Leroi, from this evidence, decides in favour of the possibility, but against the utility of lithotripsy in young children, unless we are assured that the stone is small. We would go farther, and decide against the operation in all cases. It is not so much the size of the stone as the nature and the size of the instruments that we must consider. It is the *operation* which is inapplicable to the small urethra of the child. It is more painful, more tedious, more uncertain, and, from the aggregate of all these circumstances, it is probably more dangerous than lithotomy. The very repetition of the manipulations is a fatal objection to the process in children. They bear pain ill, are thrown into a state of nervous excitement, and either grow unmanageable, or perhaps become affected with serious symptoms. It is a great object to get an operation done at once in children. The objections we have urged apply with much less force to lithotomy. In fact we all know that in children this operation is usually successful.

We may venture to pronounce that the question between lithotrity and lithotomy in children is, and has been long decided in favour of the latter.

2. The memoir of M. Leroi became, as we have already observed, the occasion of a discussion on the general merits of lithotrity. M. Velpeau, it would seem, first mooted this difficult point, and, supported by MM. Sanson, Souberbielle, and Pelletier du Mans, threw down the gauntlet against that operation. It was as warmly defended by MM. Amussat, Legallas, and Lisfranc. We shall not dress their arguments in the form of a debate, but state in as clear and connected a manner as possible their general tenor upon both sides.

The Report of M. Velpeau attributes, and we think with justice, the rapid progress of lithotrity, to the natural horror entertained by the world for cutting instruments.

"That which has given so much importance to lithotrity in the eyes of the world, is the fear of cutting instruments. It is the same fear that has made the fortune of caustics; of the *cura famis;* of compression in the treatment of cancers; of antiphlogistics, leeches, and divers highly praised local applications in lacrymal tumours, &c.

Is it *pain* that they pretend to avoid by lithotrity? *The operation of lithotomy occasions infinitely less!* The same is true with regard to the duration of the operation, the danger of relapse, &c. If then lithotrity is a happy conquest of modern surgery, it must remain nevertheless, a method simply exceptional, when compared with lithotomy, after human reason shall be permitted to define its natural limits. Not only in children, but in adults also, it is attended by greater inconveniences than lithotomy, whenever the stone is very hard or exceeds in size a walnut, *(gros noix)* and when the patient has not too great repugnance to the latter operation."

The two great causes which have led to the exorbitantly high estimation in which lithotrity is held, are the suppositions, grounded on the reports of the lithotritists, that its performance is attended with little pain, and that it is not a dangerous operation. Each of these points requires careful consideration.

No doubt there are some very favourable cases, in which little pain is occasioned by lithotrity. But in the great majority of instances it is a very painful operation. After a "sitting" or two the urethra and neck of the bladder are rendered sore and irritable; perhaps the mucous membrane of the latter is inflamed. The introduction of the large straight instruments is very painful, the manipulations cause much suffering, and the discharge of the fragments of the calculus is frequently attended with it also. Take a single lithotritic operation, and no doubt it is not so hard to endure as lithotomy. Nay, take a *very* fortunate case, and the whole process is not to be compared in this respect to cutting. But in the greater number of instances, the sittings are repeated, and the aggregate of the pain inflicted, is, we conceive, greater than it is in the majority of cases of lithotomy. We would not be misunderstood. In favourable cases, and with dexterous operators, lithotrity is undoubtedly a less painful operation than lithotomy. But in unfavourable cases (shall we say in most?) it is either more painful, from frequent repetition, than cutting, or, at all events, it is a moot point between them.

But is not lithotrity a very safe operation? There's the rub. Safety or danger, success or misfortune, have now become matters of experience and of demonstration, questions admitting of decision, not merely on hypothetical grounds, but on facts. Yet those facts are hard to be obtained. They are mostly in the lithotritist's keeping, and, aware of the jealousy with which he is regarded, alarmed for the fate of his infant art, can we wonder if he keeps them safely?

It is singular how widely different is the reading of the same page by persons of opposite opinions. M. Civiale has published the particulars of certain cases in which he attempted or performed lithotrity. From *his own summary*, it appears that the number of cases operated on since 1824, have been 244. Of these there were cured 236; the number of deaths was 5; and the patients who continue to suffer, though no longer labouring under stone, are 3. Thus M. Civiale's own estimate shews a mortality of only 1 in 48⅘.

This is marvellous success. M. Civiale must have displayed much more than the wisdom of the serpent, in his selection of cases for operation, to have obtained such results as these; or he must have experienced such good fortune as ordinary mortals cannot look for. It is totally unlike what any other lithotritist has met with, and when we reflect that many of the operations were performed with instruments now acknowledged to be dangerous: and that the earlier ones were resorted to at a time when the affections of the kidney were little understood in France, if indeed they are so at the present moment, we confess ourselves unable to offer any rational explanation of such triumphant results.

Let us listen, however, to M. Velpeau, and hear his deductions from M. Civiale's cases. According to this gentleman, of the 244 cases of M. Civiale, there were cured 130, and the number of patients dead *or* retaining the stone is 114!

The discrepancy between the two statements is enormous. It is true that M. Velpeau's statement is loose, but his numbers are precise, and the declaration being made in the very face of M. Civiale, it is incumbent on the latter to disprove it if he can. Being taxed with exaggeration, M. Velpeau replied:—

"At the academy, M. Civiale might have assured himself that if facts have been altered, it has not been by me; and that I am also acquainted with the memoir which he has inserted in the fasciculi of the academy, and it is precisely because he has taken the precaution to publish all his observations, that I have arrived at results in figures so different from his."

Leaving this particular question for the present we proceed to some other statements made by M. Velpeau.

"M. Velpeau proceeded to state the accredited results, upon which were based his conclusion that the mortality from calculus in the hands of the friends of lithotripsy was greater than followed in the hands of the lithotomists. From this statement it appears that neither M. Amussat, M. Leroi d'Etoilles, nor Heurteloup have ever published complete accounts of their results, and their success or failure in selected cases should weigh but little in a general question. There remain the summaries of MM. Civiale and Bancal, together with certain private information communicated by M. Leroi, but which M. Velpeau did not feel warranted in stating, and some data of more ancient date. The evidence furnished by these is far from favourable in the opinion of M. Velpeau. He stated that of eighty-three cases *operated on* by the most able lithotritists, forty-two were cured, thirty-eight died, and of the forty-two cured, nineteen had serious accidents—a result which he acknowledged was 'very different from that which appears on the face of the tables, (Rapport Larrey).'"

We observed, in continuation, that of 24 cases operated on in the Hospital Necker, 14 are cured, 11 have proved fatal. Of 45 cases since operated on, 30 are cured, 15 have been fatal. In 10 other cases, the stone is retained. Of 30 cases published by Sedaire, in the Gazette des Hôpitaux, 18 are cured, 8 have been fatal, and in 4 the stone remains.

It is obvious that these statements are not to be entirely trusted in a statistical inquiry. They may be picked. But if the lithotritists object to this evidence, as they well may, it is necessary for them to produce the whole truth. We much fear that the acrimony of the present discussion will tend to the very opposite effect, and that the day has not yet arrived when authentic and uncoloured facts can enable us to arrive at unprejudiced conclusions.

Yet, although we think it would be unsafe to generalise on such data as M. Velpeau has brought forward, they will tend to convince the most sanguine advocates of lithotrity, that it cannot be regarded as a trivial operation, nay, that we must pause before we admit it to be a very successful one. The deaths are many, the failures, independent of death, are not few, and the balance is more even between the operations of lithotomy and lithotrity, than many have supposed, or than some will still allow. It is absurd at present to attempt an estimate of the mortality of the latter, but it is right that what is actually known should be widely known, in order to disabuse the profession at large of extravagant and erroneous notions.

We will now revert to the discussion between MM. Civiale and Velpeau, with respect to the 244 cases of the former. We do so because it involves a principle of no little consequence—not for the sake of the personal difference between those gentlemen. We extract the following passage from our contemporary.

"As it regards the question at issue with M. Civiale, it seems that he has selected from the cases reported by the latter surgeon, two hundred and forty-four observations in which the operation of lithotripsy was, *in his opinion*, performed. The character of the commentator is a sufficient guarantee for the fact, that of *these* two hundred and forty-four cases, but one hundred and thirty were *cured*. But there is an important breach in the chain of evidence. We are no where exactly informed what M. Civiale regards as a lithotritic operation, nor are we made certainly acquainted with the circumstances which he considers as a sufficient proof of cure. M. Civiale has defined what *he* considers an operation. 'I have said, in the last faciculus of tho academy, where the exploration terminates, and where the operation begins.'—*Letter to the Academy*.

The last fasciculus has not yet reached us, and we can only infer the opinion of M. Civiale, from the following passage in his letter, and from the remarks of M. Velpeau; but the inference is very plain.

'As to the pretension which has been advanced in some writings expressly designed to depreciate lithotripsy; to consider as real operations the preliminary examinations that are necessary to determine the condition of the patient, and to ascertain whether the operation can or cannot be performed, if any thing could surprise us, it is that this pretension should again make its appearance in the academy.' *loc. cit.*

It is evident, from these passages, and the conflicting statements of the parties, that the two hundred and forty-four cases of operation, chosen by M. Velpeau, from the returns of M. Civiale, even if the acknowledgment of the former to that effect had been wanting, are not the same two hundred and forty-four cases acknowledged to have undergone the operation by the latter surgeon. We have then two series of observations re-entering and becoming confounded with each other at various points, and the conclusions drawn from one series can be of no value in estimating the accuracy of the other; the very attempt to use them for such a purpose is to a certain extent unfair.

The next question of any interest to the profession, is the value of the tabular result of each of these series considered separately, as elements of statistical calculations. That of M. Civiale's report, hangs on the validity of his definition of a lithotritic operation, and the nature of the manipulations exercised upon those patients whose cases fall without the definition. Let us hear M. Velpeau on this subject.

'As for myself, I have made use of the facts published by M. Civiale, thinking it impossible to draw them from a better source. Nevertheless, seeing that we build on the same foundations, how comes it that we differ so widely in our calculations? M. Civiale positively refuses to admit, that the examinations, the attempts that are made to recognise the existence of the stone, to seize it, or to break it with instruments, should be considered as operations. This is an idea that we find in all his writings. Let us see, now, the nature of these preliminaries. The litholabe, the brise-pierre, or the percussor, are introduced into the bladder, where one extremity is made to move about, in order to ascertain the existence and the situation of the stone. Then the instrument is opened; the branches are separated, to seize or embrace the foreign body, and to ascertain its dimensions and its form. Finally, an attempt is made to perforate, to crush, or to break in pieces the stone, by acting on the other extremity of the lithotritor, which is large and straight, in the urethra. It is repeated one, two, or three times, at intervals of a few days. It may now, perhaps, be demanded of me, in what respect the operation differs from these preliminaries. Truly, I know not! It has always appeared to me that, *once in the bladder*, the instruments expose the patient to as much danger when they manœuvre in the empty space, or fruitlessly, as when they act with real efficacy upon the calculus.'—*Lettre de M. Velpeau.*"

It is impossible, we think, for any practical surgeon to agree with M. Civiale. It is very well to insulate the mere crushing of the stone, from those manipulations which were necessary in order that it may be crushed, and tell us that *that* alone constitutes the operation. Such special pleading may do for the Sorbonne, perhaps for the Academy, but to the patient, we imagine, it will be any thing but satisfactory. What would be *his* gratification if he were told:—"Lithotrity is not a painful operation; but, of course, I am talking only of the crushing. It is not a dangerous operation, but still we must confine the observation to the crushing. It is true, you will suffer a good deal from the examinations, they may be ineffectual, they may terminate in your destruction, but then you cannot lay the blame to the crushing. They are not required in lithotomy, and they are requisite for lithotrity; yet if you die from *them*, you have the consolation of knowing that it is not, strictly speaking, lithotrity which kills you, and *it* remains as triumphant as ever."

Such arguments may be very good to the patient, they may be perfectly convincing, but they ought to be fairly and openly stated. For our own parts we must own, it is the mortality that seems to us the important thing, and whether the man dies at this stage or at that, from the litholabe or the percussor, is comparatively immaterial, if he dies at all. And such is the common-sense view of the case which, we fancy, will be generally taken.

Our contemporary, in his remarks upon the question, remarks with which, for the most part, we quite coincide, introduces the results of some lithotritic operations performed in America.

" Of fourteen cases treated by lithotripsy by surgeons of our acquaintance, one died by causes totally foreign to the operation ; one was subjected to an attempt which failed, owing to the excessive pain it occasioned, and the patient was afterwards cured by the lateral operation ; and twelve were cured."

Eleven other operations, performed by Dr. Randolph, of Philadelphia, were all successful.

The following are the conclusions deduced by our contemporary from the facts and arguments of the speakers in the Academy.

" 1st. It is conceded on all hands that when the bladder and urinary passages are in a healthy condition ; when there is no contra-indication from visceral or other disease ; when there exists but one stone, and this is not very hard or rugose ; when the calculus is not larger than a walnut, the patient being an adult, and when the operator has great mechanical skill combined with professional tact, and a thorough acquaintance with the operation, in preference to lithotomy, lithotripsy—*ought to be attempted.*

2d. If there be a plurality of calculi, within certain limits, the other circumstance remaining the same, lithotripsy is equally proper. This is a corollary of the last proposition.

3d. That in all cases, where the urinary organs are healthy, or nearly so, and when the simple methods of sounding employed before all operations in calculus fail in detecting an unusual hardness or size of the stone, it is warrantable to employ lithotritic instruments in the endeavour to ascertain its character.

4th. That examination by lithotritic instruments may render a patient insensible, (Roux,) or may involve his life, (Velpeau ;) hence the comparison of equality made by M. Rouchoux, between this species of examination and simple catheterism, is unjust in the highest degree.

5th. That the slightest operations in surgery, such as staphyloraphy, or suture of the perineum after rupture in delivery, *may* produce fatal consequences ; hence simple sounding is not *altogether* devoid of danger. *(Roux.)*

6. That the operation of lithotripsy is sometimes attended with so little pain, as to be likened, by a patient who had been frequently subjected to it, to the extraction of a tooth. *(Amussat.)*

7th. That it may be regarded as questionable, whether the average amount of pain suffered in operations for lithotripsy, employed on the recent extensive scale, is in any degree less than that of lithotomy.

8th. That it is *at least* questionable, whether the serious accidents following lithotripsy are not more numerous than those following lithotomy.

9th. That it is not possible to determine with absolute certainty the completion of the cure in lithotripsy, but the proof of the removal of the last fragment may amount exceedingly nearly to certainty. *(Lisfranc.)*

10th. That the dangers of the operation may be much diminished by previous general treatment in some cases, and the pain may be sometimes lightened by previous catheterism, but this is not always the case, for the tenth session may be more painful than the first. *(Ib.)*

12th. That it is impossible to determine by any statistical accounts now on record, the relative importance or success of the two methods, or to determine the same question by the first method proposed in the report.

13th. That the objection advanced by M. Amussat against the second method of ascertaining this point ; namely, the cruelty and inhumanity of the test, is a petitio principii, and devoid of weight.*

* The methods alluded to are these :—First, by comparing the mortality of

14th. That a comparison of success between an equal number of grave cases of calculus treated by the two methods, would be an injustice to the claims of lithotripsy; that a group of mild cases similarly employed would produce results unjust to lithotomy, and if a group of cases of a middle character were chosen, which would be accomplished with difficulty, the result would be an approximation to truth, dependent for its value upon the multitude and minuteness of observations *faithfully reported*."

Enough, we think, has been advanced, to show that anything like an accurate comparison, between the success of lithotomy and lithotrity, is at present unattainable. The data on which such a comparison must be formed are not in our possession.

We will go, however, farther than this, and express our conviction that no such comparison can ever be drawn, because the observations are not really applicable to the same cases. For—

1. Lithotrity is not adapted for children, in whom stone in the bladder is frequent.

2. It is not adapted for the oxalate of lime or any very hard calculus.

3. It is not adapted for very large calculi.

4. It is not adapted for cases of calculus of any size in which the bladder is diseased, or in which the urethra has undergone any material contraction.

When we sum up these cases forbidding, or, at the best, exceedingly unfavourable to the operation of lithotrity, we shall probably find that they form a large proportion, if not the majority, of those which occur in hospitals. The lower class of persons, whose sensibilities are not usually acute, and who have to gain their livelihood by labour, seldom apply for medical advice until the complaint has existed for some time, and the calculus has either attained large dimensions, or the bladder or kidney is more or less diseased. We leave then the majority of cases for lithotomy, and we leave the *worst*. So far is lithotrity from being free from danger, that it abandons the bad cases to the knife. This is a truth that should be generally known. The direct advantages accruing from lithotrity have been greatly overrated, are, perhaps, overrated now, while, singularly enough, the indirect advantages are not sufficiently understood or prized.

Lithotrity has already done much for humanity, because in the first place it has led persons, with stone in the bladder, to apply earlier for medical advice, from the belief that the operation is not a severe one; in the second place, it has enabled us, by its improved modes of sounding, to detect small stones more readily than formerly; and, in the third place, it has given us the means of removing those stones, *while small*, with comparative facility and safety.

If lithotrity is inapplicable to large stones, it finds them out before they are large, and it removes them before they become so. This is the real value of the operation, the positive benefit it confers upon man. And no mean benefit. Who that had a small calculus in his bladder would be cut for it? What professional man would dream of suffering lithotomy in his own case? None, we are sure, aware of all the facts, and of the actual state of the question.

In the middle and the upper classes, where symptoms soon attract attention, and means admit of the patient procuring good medical advice, small calculi are constantly discovered, and are now commonly removed by surgeons in this Metropolis. The bladder-forceps are introduced, after the bladder is injected with warm water, and the same instrument may sound the patient, detect the calculus, and either remove it whole, if it is small enough to be withdrawn through the urethra, or crush it by the screw or the hammer if it is

---

calculus before and since the introduction of lithotripsy. "The work of M. Blandin, who alone has had the temerity to do so, proves already that in this point of view, experience pleads incontestably in favour of lithotomy." Secondly, by comparing the results of two equal groups of selected cases, in circumstances as nearly similar as possible, but treated on the two contending systems.

not.* How trivial, how simple, how safe is the operation, when weighed against lithotomy. When once the public grow well aware of the advantage of attending to the symptoms of stone, and to the benefit resulting from early medical attendance, we may anticipate that the operation of lithotomy, as well as lithotrity, under unfavourable circumstances, will be in a great measure superseded. This article has run to so great a length, that we must drop the subject for the present. We shall return to it at a future opportunity, and present some cases in illustration of the remarks that we have offered.

---

Case of Dropsy, with Ovarian Tumors, in which the Operation of Tapping was performed Fifty Times, by William Coulson, with an Analysis of the Fluid by Dr. Bostock, V.P.R.S.

Sarah Price, æt. 38, servant, single, applied to me in April, 1833, for an enlargement of the abdomen;—says, that two or three years ago, after an attack of diarrhœa, attended with pain in the stomach, a swelling took place on the lower part of the left side. The enlargement slowly increased, and for some time was not attended with any inconvenience to herself in her occupation. Several medical men were consulted, and there was a contrariety of opinion, some thinking the enlargement caused by an ovarian tumor, others by a collection of fluid. Her pains at last became severe, and the weight and sense of suffocation so distressing, that it was determined to introduce a trocar to see if there was fluid, and on the 10th of April, 1833, this operation was performed in the most prominent part of the swelling, midway between the umbilicus and crista ilii on the right side, but no fluid escaped. The swelling continued to increase, and the sense of weight and difficulty of breathing became more distressing. There was a distinct sense of fluctuation, and, on a consultation, it was determined on the 28th of May, 1833, again to introduce a trocar. This was done a little lower than on the former occasion, and more towards the left side, when 35lbs. of fluid were drawn off. From this period to the time of her death, the fluid constantly accumulated, and when the symptoms produced by the accumulation were so severe as to prevent her attending to her occupation, the operation was repeated;—after the water was drawn off a tumor could be very distinctly felt, of the size of a child's head. With the exception of the first two or three operations, she suffered very little after the tapping. She would get up the second day, and walk the distance of a mile to her place.

The time between each operation varied, but on the whole it gradually decreased. During the last ten weeks her strength began to fail—she was scarcely able to walk, became thin, suffered severe pain—her bowels, though relaxed at first, were afterwards confined, and on the 20th August she expired.

The body was examined within a few hours after death in the presence of Dr. Thompson and Mr. Langstaff.—The right side was almost entirely filled by the enlarged ovary which adhered to the liver, the peritoneal covering of which was very much thickened: the ovary, on being cut into, presented numerous cells or cysts, of different size, some filled with a clear fluid, others with a substance like putty. The walls of the cysts were not only very thick but vascular, and the outer tunic of the ovary was also very dense and vascular. In one of the cysts there was an opening which communicated with the left side of the abdomen. The greater part of the left side was formed

---

* Weiss's bladder-forceps are those generally used. It is always better to employ one provided with a screw, because we cannot always be quite sure beforehand of the dimensions of the calculus; and if, seized by forceps unprovided with the means of crushing it, the size should not admit of its withdrawal through the urethra, it might not be easy to disengage it from between the branches of the instrument.

into a cavity in which the water collected, and was lined by a dense and vascular membrane. The peritoneum generally was inflamed and thick—the right ovary and uterus were sound; no morbid appearances were observed in any other part.

The total quantity of water drawn off by 50 operations was 1542 pounds.

*Dr. Bostock's Analysis of the Fluid.*

" I received from Mr. Coulson two specimens of a fluid taken from an abdominal tumor, one of them at the commencement, the other at the end of the operation. The former was perfectly transparent, the other contained a few floating films, and was slightly tinged with blood, but upon standing for 48 hours, the extraneous matter subsided, so that I could not perceive the least difference between the two. The fluid was transparent and perfectly homogeneous; it had a slight yellow tinge; it was readily mixable with water, and had a sp. gr. of 10058. A portion of it was evaporated at a temperature not exceeding 250°; a colourless transparent film was obtained, with a little brown matter on the margin, and with a number of imperfect cubical crystals dispersed through it—it constituted 2.2 per cent. of the entire fluid. A portion of the fluid was exposed for some time to the temperature of boiling water, and was rendered slightly opaque. The appropriate tests for albumen indicated the presence of this substance, but only in minute quantity. The residuum was found to consist partly of saline and partly of animal matter, in very nearly equal quantity. Nearly the whole of the saline matter was muriate of soda; —by the action of nitrate of silver it was found to constitute as nearly as possible 1 per cent. of the entire fluid. There was a slight excess of soda, just sufficient to affect a test paper, and an indication of phosphoric acid, but they were in too minute quantity to enable me to ascertain their proportion.

With respect to the animal matter, which constituted rather more than half of the residuum, or about 1.2 per cent. of the entire fluid, I have stated above, that it contained albumen, so as to be recognized by the appropriate tests, but in small quantity only. I likewise obtained from it what I have termed albumino-cerous matter—a substance which I am disposed to believe will be generally found to exist in fluids that have been, for any length of time, retained in close cavities. The greatest part of it, at least ¾ of its whole weight seemed to be composed of the uncoagulable or muco-extractive matter, which constitute the principal part of the animal ingredient of the serosity of the blood—a substance which, in its chemical properties, I believe is nearly related to urine.

The following may be regarded as approximating to the constitution of the fluid under consideration:—

| | |
|---|---|
| Water .. .. .. .. .. | 978 |
| Muriate of soda .. .. | 10 |
| Muco-extractive .. .. | 9 |
| Albumino-cerous .. | 1 |
| Residua of albumen, soda, phosphoric-acid, &c. .. .. | 2 |
| | 1000" |

### On Mercury in Onychia Maligna. By Dr. Beck, of New York.

Mr. Wardrop published a very accurate description of onychia maligna in the Medico-Chirurgical Transactions many years ago, and he there remarks that, in the treatment of this complaint, the only local means which he found to afford relief, were the evulsion of the nail, and the subsequent application of escharotics to the ulcerated surface.—Even this treatment does not always succeed, and amputation is often necessary. He then tried mercury, and, as soon as the system came under its influence, the ulceration assumed a healthy appearance, and the bulbous enlargement began to subside. Dr. Beck has been able to confirm Mr. Wardrop's observations, and has published some cases with that view.

The following description is short, and may be introduced here. Mr. Wardrop loquitur.

"The commencement of the disease is marked by a degree of swelling of a deep red colour of the soft parts at the root of the nail. An oozing of a thin ichor afterwards takes place at the cleft formed between the root of the nail and soft parts, and at last the soft parts begin to ulcerate. The ulcer appears on the circular edge of the soft parts at the root of the nail; it is accompanied with a good deal of swelling, and the skin, particularly that adjacent to the ulcer, has a deep purple colour; the appearance of the ulcer is very unhealthy, the edges being thin and acute, and its surface covered with a dull yellow or brown-coloured lymph, and attended with an ichorous and very fetid discharge. The growth of the nail is interrupted; it loses its natural colour, and at some places appears to have little connexion with the soft parts. In this state," he adds, "I have seen the disease continue for several years, so that the toe or finger became a deformed bulbous mass. The pain is sometimes very acute, but the disease is more commonly indolent, and accompanied with little uneasiness: this disease affects both the toes and the fingers. I have only observed it on the great toe, and more frequently on the thumb, than any of the fingers. It occurs, too, chiefly in young people, but I have often seen adults affected with it."

Of the cases occurring in Dr. Beck's practice, four were ten years of age or under—one of the age of 23. In one case the disease affected the thumb—in a second the great toe—in the others the fingers. They seldom complained of much pain. We shall make room for one case in illustration.

"Decatur Frazer, a boy ten years of age, in July, 1821, ran a small splinter, by accident, under the nail of the right thumb; in a few days the parts about the root of the nail assumed a livid colour, and at the same time began to swell, and afterwards to ulcerate. From this time the swelling gradually increased, and the discharge became more copious and offensive. In this state it continued until about the beginning of January, 1822, a great variety of local applications having been made in the mean time, without the slightest advantage. At this time the little patient was placed under the care of my friend, the late Dr. James Kent Platt, Professor of the Institutes of Surgery in the University of Vermont. The extremity of the thumb had assumed a bulbous appearance, and was about double the natural size. The nail had separated in the middle and was quite loose. The matter discharged from the ulcer was thin and extremely offensive; so much so as to require frequent dressings during the day. There was no pain complained of, except a slight sensation of it whenever the ulcer was cleansed and dressed. Judging it to be a case in which mercury might prove serviceable, the blue-pill was commenced with. Several of these were taken, but from the negligence of the parent of the boy, without any regularity. No effect was produced upon the gums, and no improvement visible in the appearance of the thumb. Dr. Platt being obliged to visit the West Indies for the benefit of his health. I was requested to attend the patient. I continued him under the use of the blue-pill for a few days, but without any apparent advantage. The mother now became impatient, and declined giving any more mercury for the present. After an interval of five or six weeks I resumed the use of mercury in the form of pills composed of calomel and opium in the proportion of one grain of the former to one-eighth of a grain of the latter, three times a day. In about ten days the mouth became affected, and a pretty free salivation ensued. Almost instantaneously upon this effect being produced, the ulcer put on a different appearance. In a few days the discharge from it ceased completely, and in about three weeks the whole had healed, and the swelling almost entirely subsided. Between two and three months after this a new nail had grown out, and the thumb has remained healthy and sound to the present day."*

* Researches on Medicine. By Dr. Beck.

### LITHOTOMY A DEUX TEMPS, ON A CHILD.*

The following case is related in the India Journal, which, we are happy to observe is taking root, and thriving amidst our Indian brethren. It is likely to be of service to the profession in that quarter, for it stimulates the indifferent to exertion, and affords opportunities to the zealous for distinction. It is conducted by Mr. Corbyn with professional ability and with gentlemanly feeling, both, after all, indispensable requisites for the permanent success of any periodical, addressed to a liberal and enlightened class of readers.

*Case.* A delicate and irritable Moosulmann child, ætatis 7, had suffered from the stone for two years, and was greatly reduced in consequence. The boy was taken in quite a desperate state to Mr. Brett, at Cawnpoor, in November, 1834. At the earnest solicitation of the father, and as a last resort, lithotomy was performed by Mr. B.

"The straight staff and scalpel were the only instruments employed, and there was no difficulty until the introduction of the forceps. The lower end of the stone was repeatedly seized, and as often escaped from the grasp of the forceps, being evidently retained either by the firm contraction of the bladder, or by a portion of it being encysted. Finding my efforts unavailing and the child becoming much exhausted, the pulse rapid and small, and profuse perspiration, I desisted from further efforts, administered a full dose of tincture of opium, combined with aromatic spirit of ammonia, and sent him to bed,—constant fomentations being applied. The above measures had the most happy effect. The child rallied, the anodyne and soothing treatment was persevered in, and the ensuing morning the stone was removed in the following manner. An assistant seated on a chair placed the patient on his lap with his back towards him, bent and raised the knees by introducing his arms under the hams, the perineum being lowered as much as possible, the body kept in the erect position, so that the stone might gravitate as much as possible towards the lower part of the wound. Introducing my finger, I felt the calculus which was immediately seized by the forceps and withdrawn. One extremity of the calculus had been lodged in a *cul-de-sac* of the bladder, as was evident by the form of the stone being of smaller diameter than the rest, and having a clearly defined neck, where it had been compressed apparently by the separated fibres of the muscular coat of the bladder, which with the spasmodic action of the bladder in so irritable a subject, satisfactorily accounts for the impediment. I have not the slightest doubt that had I persisted in my efforts to remove the stone, the child would have expired on the table, and the expediency of procrastination on the occasion is thus exemplified. Difficulties in Lithotomy do sometimes occur when least anticipated, requiring all our self-possession and deliberate reflection, and these occasional difficulties, I dare say, gave occasion to the exclamation of Abernethy. 'Oh! egad, 'tis an horrible operation. You don't know what you may be doing.'"

No doubt, Mr. Brett was perfectly right in adopting the practice that he did in this instance. Perhaps the stone is too frequently removed at the time of the operation, from a feeling of natural yet false shame in the operator. It must be owned, however, that the occasions which really require the stone to be left are rare, more rare than the cases in which that proceeding has been had recourse to. A surgeon may pass a life-time and not meet with an instance of the sort.

* India Journ. March 1, 1836.

